# Global research trends and hotspots on tendon-derived stem cell: a bibliometric visualization study

**DOI:** 10.3389/fbioe.2023.1327027

**Published:** 2024-01-08

**Authors:** Songou Zhang, Jinxiang Shang, Zhiqian Gu, Xiaopeng Gu, Fei Wang, Xujun Hu, Guoliang Wu, Huan Zou, Jian Ruan, Xinkun He, Chenzhou Bao, ZhenYu Zhang, Xin Li, Hong Chen

**Affiliations:** ^1^ Department of Clinical Medicine, Health Science Center, Ningbo University, Ningbo, Zhejiang, China; ^2^ Department of Orthopedics, Affiliated Hospital of Shaoxing University, Shaoxing, Zhejiang, China; ^3^ Department of Orthopedics, Shaoxing People’s Hospital, Shaoxing, Zhejiang, China; ^4^ Department of Orthopedics, Ningbo Sixth Hospital, Ningbo, Zhejiang, China; ^5^ Department of Clinical Medicine, School of Medicine, Shaoxing University, Shaoxing, Zhejiang, China

**Keywords:** tendon-derived stem cell, tendon injury, bibliometric analysis, scaffolds, mechanism, inflammation

## Abstract

**Purpose:** This study was aimed to examine the global research status and current research hotspots in the field of tendon stem cells.

**Methods:** Bibliometric methods were employed to retrieve relevant data from the Web of Science Core Collection (WOSCC) database. Additionally, Citespace, Vosviewer, SCImago, and Graphad Prism were utilized to analyze the publication status in this field, identify the current research hotspots, and present a mini-review.

**Results:** The most active countries in this field were China and the United States. Notable authors contributing significantly to this research included Lui Pauline Po Yee, Tang Kanglai, Zhang Jianying, Yin Zi, and Chen Xiao, predominantly affiliated with institutions such as the Hong Kong Hospital Authority, Third Military Medical University, University of Pittsburgh, and Zhejiang University. The most commonly published journals in this field were Stem Cells International, Journal of Orthopedic Research, and Stem Cell Research and Therapy. Moreover, the current research hotspots primarily revolved around scaffolds, molecular mechanisms, and inflammation regulation.

**Conclusion:** Tendon stem cells hold significant potential as seed cells for tendon tissue engineering and offer promising avenues for further research Scaffolds, molecular mechanisms and inflammation regulation are currently research hotspots in this field.

## 1 Introduction

Tendons are fibrous connective tissues that connect muscles to bones. They are characterized by avascularity and cellular scarcity, which limits their self-repair capabilities, frequently resulting in the formation of scar tissue during the healing process, reducing tendon flexibility and increasing the risk of re-rupture (Andarawis-Puri et al., 2015; Thomopoulos et al., 2015; Millar et al., 2021; Pearce et al., 2021). Restoring the normal structure of injured tendons poses a significant challenge in sports medicine. Tendon-derived stem cells (TDSCs) are a type of mesenchymal stem cell found within the tendon tissue. Strictly speaking, TDSCs cannot be classified as conventional stem cells due to their biological heterogeneity. It is more accurate to describe them as “stem/progenitor” cells considering their capacity to differentiate into a limited number of specific cell lineages. Besides, they possess certain stem cell features such as clonogenicity, high proliferation rate, and self-renewal ability (Bi et al., 2007). We summarize cell culture methods reported in TDSCs research in Supplementary Table S1. Simply put, the methods for culturing and isolating tendon stem cells are as follows: under aseptic conditions, tendon tissues are treated with collagenase (usually Type I or II, at a concentration of approximately 0.1%–3%) at 37°C for several hours to overnight to isolate the cells. The cells are then collected and cultured in a specific medium (such as low-glucose DMEM), with 10%–20% serum added for nutrition, and maintained at 37°C in an environment with 5% CO_2_, with passaging done at appropriate intervals to maintain cell viability. TDSCs are characterized by the presence of markers such as CD44, CD146, CD105, and CD90, which are typical of mesenchymal stem cells (Zhang and Wang, 2010a; Lee et al., 2018). Due to their unique cellular microenvironment, TDSCs have a greater capability to generate tendon and joint tissue compared to bone marrow-derived mesenchymal stem cells (BMSCs) (Tan et al., 2012). The current cell sources of research on TDSCs are mainly: rat, mice, rabbit and human; a small amount of TDSC studied are from horse, pig. The main research focuses are: therapeutic targets and drug effect, disease mechanisms, tissue engineering, and cell properties. (Supplementary Table S1)

After a tendon injury, successful restoration of tendon integrity involves three stages: the inflammatory phase, cellular proliferation phase, and extracellular matrix (ECM) reconstruction phase. In the inflammatory phase, it involves the infiltration of inflammatory cells, secretion of inflammatory factors, and recruitment and activation of TDSCs (Vinhas et al., 2018; Ackerman et al., 2021). The cellular proliferation phase is characterized by the generation of new tendon cells, while the ECM reconstruction phase involves the formation of new ECM and tendon structure. TDSCs play a crucial role in tendon repair by secreting ECM specific to tendons and differentiating into tendon cells (Zhang et al., 2019a). Activating endogenous tendon stem cells or transplanting TDSCs using appropriate techniques has emerged as an innovative approach to promote tendon injury repair (Lee et al., 2015). Therefore, TDSCs hold significant potential in enhancing the healing of tendons and tendon-bone junctions (Chen et al., 2013).

The importance of TDSCs in orthopedic research has led to a considerable amount of research in recent years (Leong et al., 2020). However, most studies have focused on specific aspects of TDSCs research, resulting in a lack of comprehensive analysis of the literature in this area. A particular article claims to employ bibliometric methods to study TDSCs (Long et al., 2022); However, its literature search content was inaccurate. Although the discovery of TDSCs dates back to 2003, the selected literature in that study included a substantial number of publications prior to 2003. A thorough examination of its search methodology revealed that the chosen literature mainly concerned adipose-derived stem cells (ADSCs) and BMSCs in the context of tendon injury research, with limited relevance to TDSCs. As a result, their research primarily reflects the involvement of stem cells in tendon injuries (Long et al., 2022). Therefore, it is essential to utilize appropriate methods to investigate the global knowledge framework, research frontiers, and hotspots in the field of TDSCs research.

Bibliometrics is a research methodology that employs mathematical and statistical techniques to explore the fundamental aspects of scientific research (Shang et al., 2023). Recently, this method has gained widespread usage in scientific research to identify research hotspots and future directions in specific fields. However, literature searches have not produced any accurate bibliometric studies reflecting the current status of TDSCs research. Consequently, a new bibliometric study is necessary to unveil the authentic state of research in this field. This study utilizes software tools such as VOSviewer and CiteSpace to analyze trends and hotspots in TDSCs-related research.

## 2 Methods

### 2.1 Search strategy

A literature search focused on TDSCs was conducted using the Web of Science Core Collection (WOSCC) database (https://www.webofscience.com/wos/woscc/basic-search). The search strategy incorporated various title search terms, including “tendon stem cell*", “tendon derived stem cell*,” “tendon progenitor cell*,” “tendon stem/progenitor cell*,” “tendon derived stem/progenitor cell*,” and “tendon progenitor/stem cell*.” The search was limited to articles from the Science Citation Index Expanded (SCIE) database, written in English, and comprising original articles and reviews. The retrieved content included article titles, authors, abstracts, keywords, and all citations, which were downloaded in plain text format. The search was concluded on 12 July 2023.

### 2.2 Data extraction and bibliometric analysis

**FIGURE 1:**
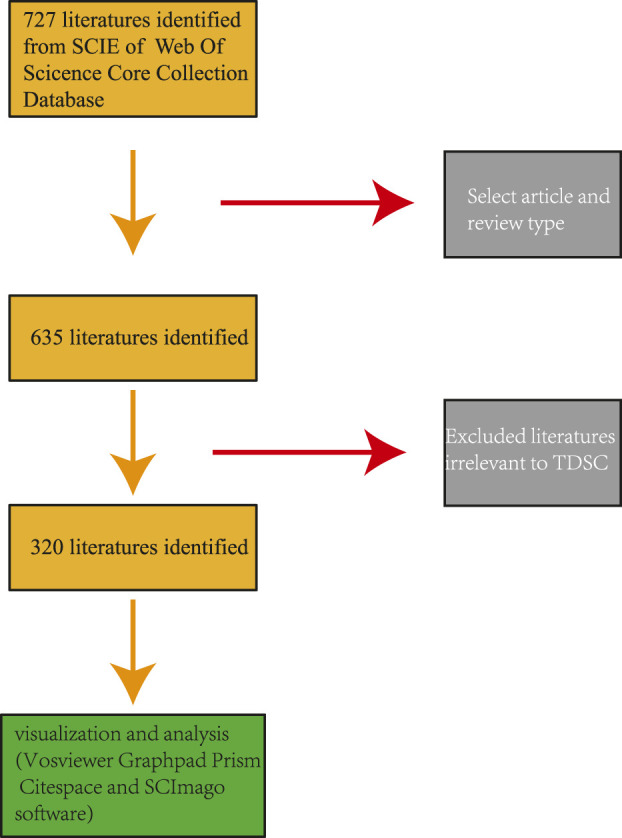
Publication screening flowchart.

Following the literature search, abstract readings were performed by SongOu Zhang to select relevant articles on TDSCs while excluding studies on mesenchymal stem cells like BMSCs and ADSCs in the context of tendon injuries, as well as any TDSCs research unrelated to the study. Subsequently, all plain text data were downloaded, and the number of articles and the annual publication rate were recorded. To conduct literature analysis and visualization, four software applications were employed: Citespace (Ver 6.1.6), VOSviewer (Ver. 1.6.18), SCImago (Ver. 1.0.35), and GraphPad Prism (Ver. 9.0.2). (Figure 1).

## 3 Results

### 3.1 Annal publications

**FIGURE 2:**
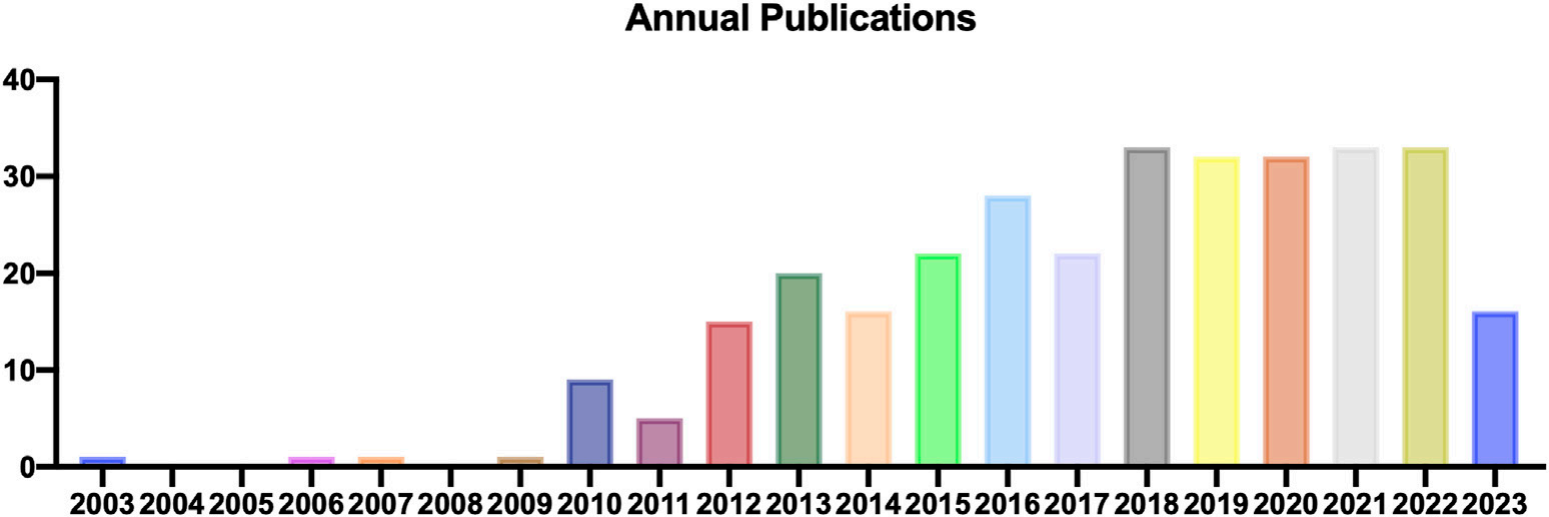
Annual output of TDSCs.

During the literature screening concerning TDSCs, we rigorously selected articles directly relevant to TDSCs and excluded those unrelated ones. Based on Citespace software, we analyzed the annual publication count and identified the earliest literature on TDSCs that published in 2003, followed by scattered articles in 2006, 2007, and 2009, and a subsequent rapid increase of articles from 2010 to 2017. From 2018 to 2023, there has been a plateau period in TDSCs research. Thus, the field of TDSCs research is relatively new and has been experiencing a growing trend in the number of studies (Figure 2).

### 3.2 Country and organization analysis

**FIGURE 3:**
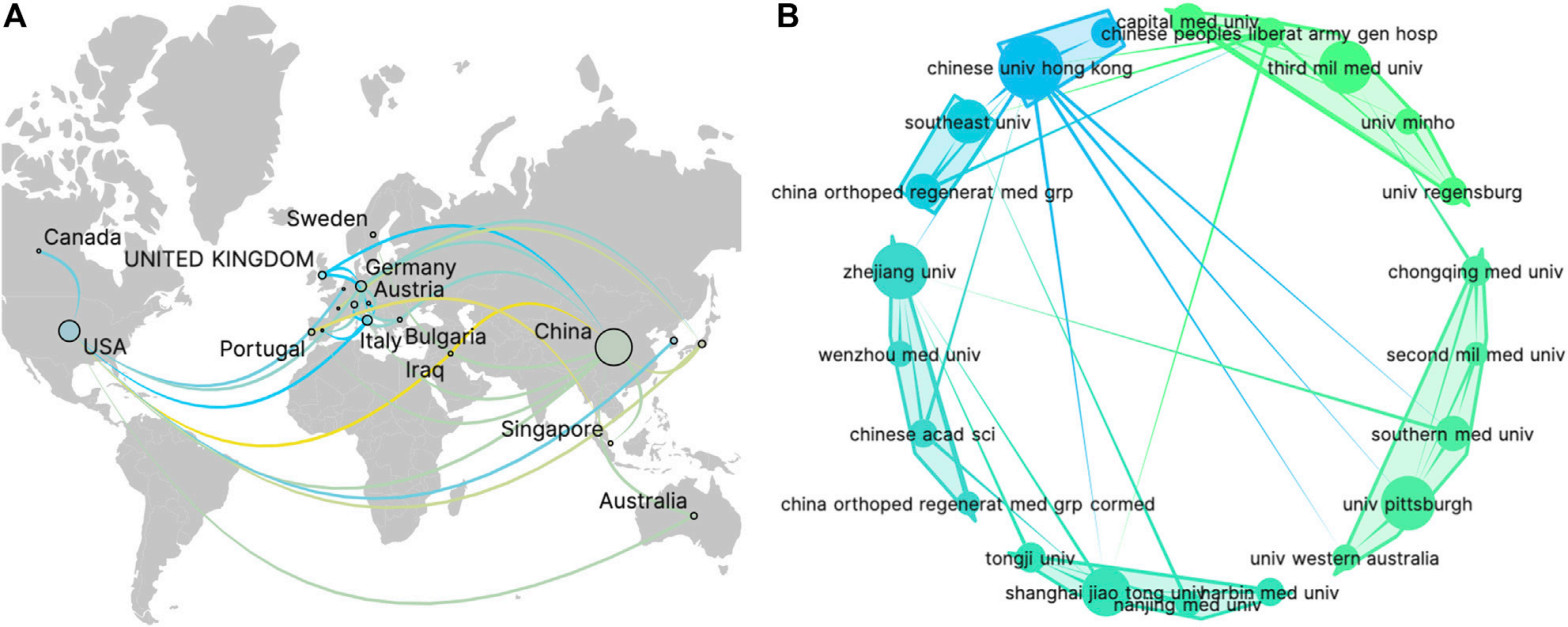
The visualization of countries **(A)** and organizations **(B)** on research of TDSCs.

Authors involved in TDSCs research came from 25 different countries. Among them, 10 countries had each published more than 5 articles in this field, including China, the United States, Germany, Italy, England, Japan, South Korea, Switzerland, and Australia. Supplementary Table S2 presents the top ten countries based on their publication numbers and corresponding citation counts. Notably, China showed the highest publication count, reaching 211, significantly surpassing other countries. China also led in citation count with 5356 citations, although the average citation per article was relatively low. On the other hand, the United States has the highest average citation per article, reaching 60.1 times per article. These findings suggest that research from China is most active, while research from the United States attracts substantial attention from authors in this field. Additionally, an analysis of country collaborations revealed that cooperation between China and the United States was the most prominent (Figure 3A).

Within this field, a total of 340 institutions have contributed to article publications. Table 1 showcases the top ten institutions based on their publication volume. Notably, nine out of these top institutions were from China, indicating that Chinese scientists are the most prolific contributors in this area. For visualization analysis, institutions that have published more than 5 articles were included, totaling 26 institutions. The visualization graph illustrates the collaborations between these institutions. (Figure 3B).

**TABLE 1:** Top 10 organizations with most publications.

Organization	Publications	Total citations	Citations/Publications	From
Chinese University Hong Kong	37	1918	51.8	China
Zhejiang University	28	1135	40.5	China
University Pittsburgh	26	1452	55.8	United States
Third Mil Med University	25	618	24.7	China
Shanghai Jiao Tong university	21	412	19.6	China
Southeast University	17	364	21.4	China
China Orthoped Regenerat Med grp	11	178	16.2	China
Capital Med University	11	150	13.6	China
Southern Med University	11	233	21.2	China
Chinese Peoples Liberat Army Gen Hospital	8	102	12.8	China

### 3.3 Author analysis

In the author analysis, a total of 1371 authors participated in publications related to TDSCs. Authors who appeared in five or more articles were included for analysis, resulting in 60 authors meeting this criterion. Table 2 presents the top ten authors based on the number of publications. It was evident that Lui Pauline Po Yee from Hong Kong Hospital Authority was the most prolific author, with a total of 24 published papers on TDSCs and 1576 citations for those papers. Both the publication counts and citation counts ranked first, indicating that Lui Pauline Po Yee is the most active author in the TDSCs field. Among the top ten authors, 8 are from China, 1 is from the United States, and 1 is from Germany.

**TABLE 2:** Top 10 authors with most publications.

Author	Publications	Citations	Citations/Publications	Organization	From
Lui Pauline Po Yee	24	1576	65.7	Hong Kong Hospital Authority	China
Tang Kanglai	21	393	18.7	Third Military Medical University	China
Zhang Jianying	19	1266	66.6	University of Pittsburgh	United States of America
Yin Zi	15	937	62.5	Zhejiang University	China
Chen Xiao	15	961	64.1	Zhejiang University	China
Zhou Mei	13	161	12.4	Third Military Medical University	China
Li Gang	13	704	54.2	The Chinese University of Hong Kong	China
Tang Hong	12	160	13.3	Third Military Medical University	China
Chen Lei	12	367	30.6	Third Military Medical University	China
Docheva Denitsa	12	556	46.3	University Medical Centre Regensburg	Germany

**FIGURE 4:**
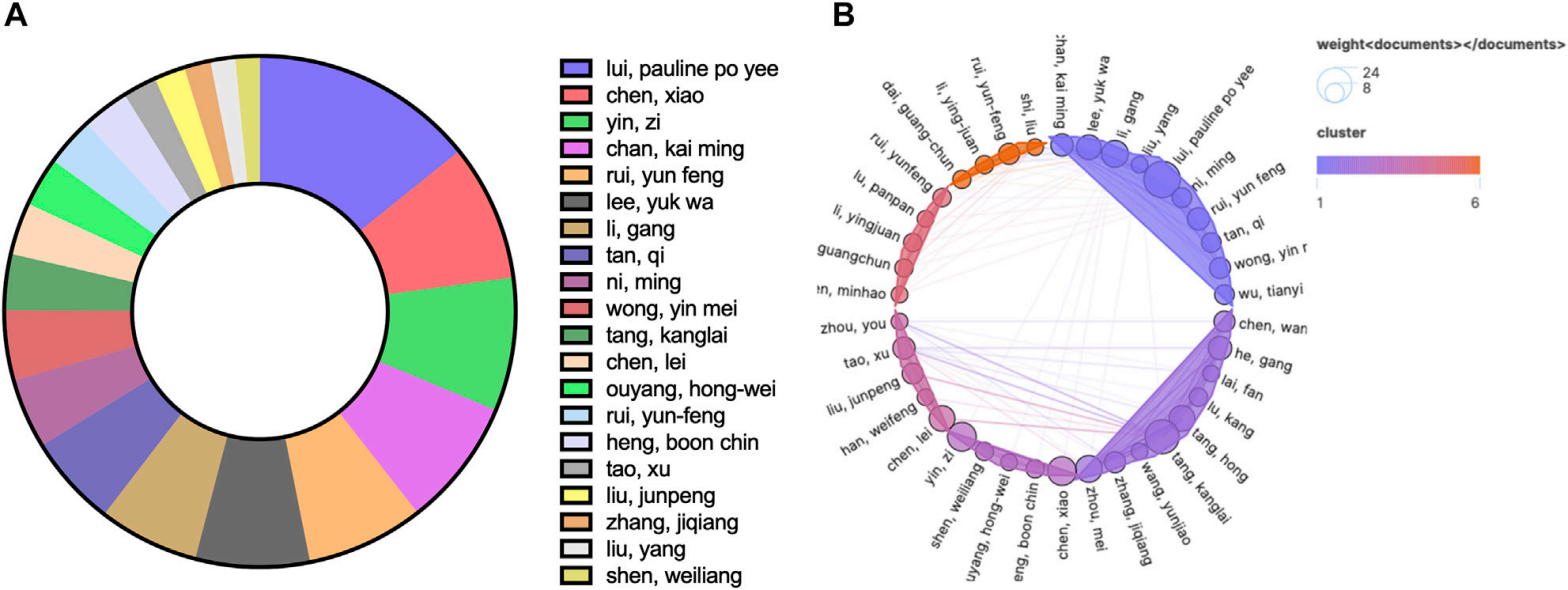
The top 20 authors by number of published articles **(A)** and their collaborative relationships **(B)**.

Figure 4A illustrates the situation of the top 20 authors based on the number of citations they received. Among them, 4 authors are affiliated with Third Military Medical University, and 2 authors are affiliated with Zhejiang University. Figure 4B displays the number of publications by each author and their collaborative relationships. The size of the circles represents the number of publications, while the colors and convex hulls indicate different clusters based on collaboration relationships. The lines connecting the circles indicate the extent of collaboration (Figure 4B). This visualization allowed us to observe the collaborative patterns among the authors.

### 3.4 Journal analysis

**FIGURE 5:**
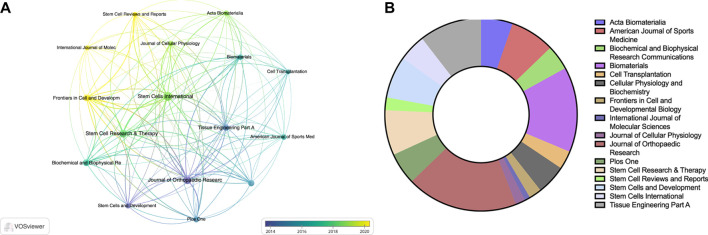
The visualization of journals **(A, B)** on research of TDSCs.

Regarding journal publications, only journals that have published more than 5 articles related to TDSCs were included in the analysis, resulting in 16 articles being included in the study. Supplementary Table S3 displays the top 10 journals based on the number of publications. Among these journals, *Stem Cells* International had the highest publication count, with a total of 19 articles. Figure 5A illustrates the status of journals focused on the field of TDSCs, where the size of circles indicates the publication volume and the colors represent the publication years. Figure 5B presents the citation counts for the relevant journals. Notably, the Journal of Orthopaedic Research has the highest number of citations, with a total of 1000. Among these ten journals, Biomaterials has the highest impact factor (IF), reaching 14.

### 3.5 Publications analysis

The analysis of publication can reflect the status of published papers in the field, including the papers that receive the most attention, the papers that appear most in the references. Out of 320 articles, there were 19 articles that have been cited more than 100 times. Table 3 presents the top ten articles based on citation count. The most cited article was “*Identification of tendon stem/progenitor cells and the role of the extracellular matrix in their niche*,” authored by Yanming Bi, with 970 citations. It reported the first identification of TDSCs from both animal and human tendons. Among the top ten cited articles, the earliest one was authored by R Salingcarnboriboon. It was cited for 194 times, focusing on the demonstration of mesenchymal stem cell characteristics in cell lines derived from tendons.

**TABLE 3:** Top 10 citations of publication.

Rank	Title	Author	Citations	Published year	DOI
1	Identification of tendon stem/progenitor cells and the role of the extracellular matrix in their niche	Yanming Bi	970	2007	10.1038/nm1630
2	The regulation of tendon stem cell differentiation by the alignment of nanofibers	Zi Yin	471	2010	10.1016/j.biomaterials. 2009.11.083
3	Characterization of differential properties of rabbit tendon stem cells and tenocytes	Jianying Zhang	260	2010	10.1186/1471-2474-11-10
4	Isolation and Characterization of Multipotent Rat Tendon-Derived Stem Cells	Yun-Feng Rui	225	2010	10.1089/ten.TEA. 2009.0529
5	Establishment of tendon-derived cell lines exhibiting pluripotent mesenchymal stem cell-like property	R Salingcarnboriboon	194	2003	10.1016/s0014-4827 (03)00107-1
6	Platelet-Rich Plasma Releasate Promotes Differentiation of Tendon Stem Cells into Active Tenocytes	Jianying Zhang	161	2010	10.1177/0363546510376750
7	Tendon-derived stem cells (TDSCs) promote tendon repair in a rat patellar tendon window defect model	Ming Ni	156	2012	10.1002/jor.21559
8	Mechanobiological response of tendon stem cells: Implications of tendon homeostasis and pathogenesis of tendinopathy	Jianying Zhang	156	2010	10.1002/jor.21046
9	Harnessing endogenous stem/progenitor cells for tendon regeneration	Chang H. Lee	153	2015	10.1172/JCI81589
10	Comparison of Potentials of Stem Cells Isolated from Tendon and Bone Marrow for Musculoskeletal Tissue Engineering	Qi Tan	144	2012	10.1089/ten.TEA. 2011.0362

In the field of this research, a total of 8752 articles have been cited. Among them, 51 articles have been cited more than 20 times. Table 4 displays the top ten articles based on the number of co-citations. Notably, the most co-cited article was “*Identification of tendon stem/progenitor cells and the role of the extracellular matrix in their niche*,” authored by Yanming Bi. It indicates that this article holds significant influence in the field.

**TABLE 4:** Top 10 Co-cited publications.

Rank	Title	Author	Publised year	Doi
1	Identification of tendon stem/progenitor cells and the role of the extracellular matrix in their niche	Yanming Bi	2007	doi 10.1038/nm1630
2	Isolation and characterization of multipotent rat tendon-derived stem cells	Yun-Feng Rui	2010	10.1089/ten.tea. 2009.0529
3	Characterization of differential properties of rabbit tendon stem cells and tenocytes	Jianying Zhang	2010	10.1186/1471-2474-11-10
4	Tendon-derived stem cells (TDSCs) promote tendon repair in a rat patellar tendon window defect model	Ming Ni	2012	10.1002/jor.21559
5	Comparison of potentials of stem cells isolated from tendon and bone marrow for musculoskeletal tissue engineering	Qi Tan	2012	10.1089/ten.tea. 2011.0362
6	Tendon-derived stem/progenitor cell aging: defective self-renewal and altered fate	Zuping Zhou	2010	10.1111/j.1474-9726.2010.00598.x
7	Mechanobiological response of tendon stem cells: implications of tendon homeostasis and pathogenesis of tendinopathy	Jianying Zhang	2010	10.1002/jor.21046
8	Uncovering the cellular and molecular changes in tendon stem/progenitor cells attributed to tendon aging and degeneration	Julia Kohler	2013	10.1111/acel.12124
9	The regulation of tendon stem cell differentiation by the alignment of nanofibers	Zi Yin	2010	10.1016/j.biomaterials. 2009.11.083
10	Analysis of the tendon cell fate using Scleraxis, a specific marker for tendons and ligaments	R Schweitzer	2001	10.1242/dev.128.19.3855

### 3.6 Keyword analysis

**FIGURE 6:**
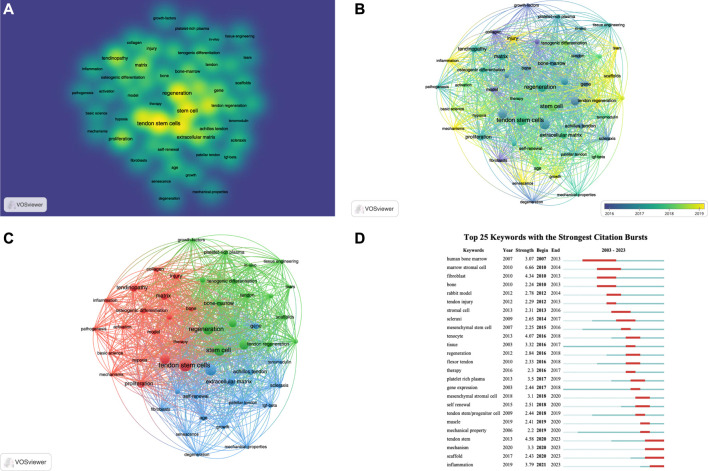
The visualization of keywords **(A–C)** and keywords bursts **(D)** on research of TDSCs.

Keyword analysis through VOSviewer and Citespace software can display the keywords with the highest frequency and the specific time when keywords appear together, and can also reflect changes in research hotspots. Regarding keyword analysis, a total of 1248 keywords were identified after merging synonyms. Among these, 51 keywords appeared more than 10 times. Supplementary Table S4 presents the top 20 keywords based on the number of occurrences. Figure 6A displays a keyword density map, where larger circles indicate a higher frequency of appearance. Figure 6B shows the keyword overlay map, with different colors representing the appearance time of each keyword. Figure 6C presents the keyword network map, where the 51 keywords are divided into 3 clusters. Cluster 1 contains 18 keywords, including activation, basic science, bone, collagen, expression, hypoxia, inflammation, injury, matrix, mechanisms, model, osteogenic differentiation, pathogenesis, proliferation, tendinopathy, tendon stem cells, tenocytes, and therapy. Cluster 2 includes 17 keywords, such as bone-marrow, growth-factors, *in-vitro*, *in-vivo*, mesenchymal stem cell, platelet-rich plasma, regeneration, rotator cuff, rotator cuff repair, scaffolds, stem cell, tears, tendon, tendon regeneration, tenogenic differentiation, tissue, and tissue engineering. Cluster 3 comprises 16 keywords, including Achilles tendon, age, degeneration, differentiation, extracellular matrix, fibroblasts, gene, growth, identification, mechanical-properties, patellar tendon, scleraxis, self-renewal, senescence, tenomodulin, and Transforming growth factor beta (TGF-beta). CiteSpace was also utilized to detect keyword bursts. Figure 6D displays the top 25 keywords with the highest burst strength. The earliest keyword burst was “human bone marrow,” the longest-lasting burst was “scaffolds,” and the most recent burst was “inflammation.” Through keyword analysis, we can identify the recent hotspots in TDSCs research.

## 4 Discussion

### 4.1 General information

The traditional viewpoint asserts that tendons only consist of tenocytes. However, in 2003, R Salingcarnboriboon et al. from Tokyo Medical and Dental University in Japan reported the presence of mesenchymal stem cells in 3 cell lines (TT-E4, TT-G11, and TT-D6) cultured from transgenic mice. These cell lines express tendon-specific genes such as scleraxis, SIX homeobox 1(Six1), EPH receptor A4 (EphA4), cartilage oligomeric matrix protein (COMP), and type I collagen. They can differentiate into tenocytes, fibrocartilage, osteoblasts, and adipocytes under specific conditions (Salingcarnboriboon et al., 2003). Subsequently, in 2007, Bi et al., 2007 extracted TDSCs with multidirectional differentiation and self-renewal ability from human and mouse tendon tissue. In 2010, similar stem cell characteristics were successfully extracted from species such as rats and rabbits (Zhang and Wang, 2010a; Rui et al., 2010). Since then, scientists have begun to focus on tendon stem cells. Over the past 15 years, extensive research has been conducted on TDSCs, which are widely recognized for their role in musculoskeletal system diseases. Due to their convenience of extraction, strong differentiation capacity, and abundant cell sources, TDSCs hold great promise for the repair of musculoskeletal system diseases (Liu et al., 2023a; Zhang et al., 2023a; Zhang et al., 2023b). This article systematically presented the current research status of the TDSCs field. We employ VOSviewer, CiteSpace, and SCImago software to analyze TDSCs literature downloaded from the WOSCC database. Our analysis included 260 articles authored by 1,052 individuals affiliated with 241 organizations from 18 countries. These articles were published in 25 journals and have received a total of 6,752 citations. Approximately 95% of the articles were original research.

Over the past 15 years, there has been a consistent increase in the number of publications in the field of TDSCs. Among the 18 countries contributing to this research, China (181%, 69.62%) ranked as the leading contributor, followed by the United States (58%, 22.31%), Germany (15%, 5.77%), Italy (8%, 3.08%), and Japan (7%, 2.69%). Notably, China had the largest number of publications and citations, with 4,460 citations for 181 articles, while the United States received 3,125 citations for 58 articles. On average, each article from China received 24.64 citations, whereas each article from the United States received 53.88 citations. Although China conducted extensive research in this field, its average attention per article was significantly lower compared to the United States.

Among the institutions involved in TDSCs research, five have published more than 20 articles. The top ten institutions with the most published articles included Chinese University Hong Kong, Zhejiang University, University Pittsburgh, Third Military Medical University, Shanghai Jiao Tong University, Southeast University, China Orthopedic Regenerative Medicine Group, Capital Medical University, Southern Medical University, and The Chinese People’s Liberation Army General Hospital. Nine out of these ten institutions are based in China, with only one from the United States. It is noteworthy that the average number of citations per article was the highest at University Pittsburgh, reaching 55.8 times.

In terms of foundational research, the most influential article in the TDSCs field is “*Identification of tendon stem/progenitor cells and the role of the extracellular matrix in their niche*” published by Bi et al. in the journal Nature Medicine. This article introduced the concept of TDSCs and provided methods for culturing and identifying them. It is also the most cited article in the field. The second and third most cited articles are “*The regulation of tendon stem cell differentiation by the alignment of nanofibers*” published by Yin Zi from Zhejiang University (Yin et al., 2010). This article used human-derived TDSCs to inoculate aligned nanofibers, which can promote the differentiation of TDSCs toward tendons. It introduced a very clever physical method to induce directional differentiation of TDSCs, which provided a broad idea for the application of TDSCs. The third most cited article is “*Characterization of differential properties of rabbit tendon stem cells and tenocytes*” published by Jianying Zhang et al. (Zhang and Wang, 2010a) The fourth and fifth ranked articles were “*Isolation and characterization of multipotent rat tendon-derived stem cells*” published by Yun-Feng Rui et al. (Rui et al., 2010) and “*Establishment of tendon-derived cell lines exhibiting pluripotent mesenchymal stem cell-like property*” published by R Salingcarnboriboon et al., 2003. These three articles all introduce methods for the acquiration and isolation of tendon stem cells from tendon tissue, as well as their characteristic features. The sixth (5th) (Zhang and Wang, 2010b) and eighth (6th) (Zhang and Wang, 2010c) most cited articles were also authored by Jianying Zhang et al. The seventh most cited article is “*Tendon-derived stem cells (TDSCs) promote tendon repair in a rat patellar tendon window defect model*” published by Ming Ni et al. (Lee et al., 2018). The ninth most cited article is “*Harnessing endogenous stem/progenitor cells for tendon regeneration*” by Chang H. Lee et al. (Tan et al., 2012). The four articles explored the application of TDSCs in tendon-related diseases. The 10th most cited article was “*Comparison of Potentials of Stem Cells Isolated from Tendon and Bone Marrow for Musculoskeletal Tissue Engineering*” by Qi Tan et al. (Tan et al., 2012), which compared the advantages and disadvantages of BMSCs and TDSCs as seed cells for musculoskeletal diseases. It concluded that TDSCs exhibited stronger multi-lineage differentiation ability than BMSCs and possessed significant therapeutic potential.

### 4.2 Hotspot and frontiers

**FIGURE 7:**
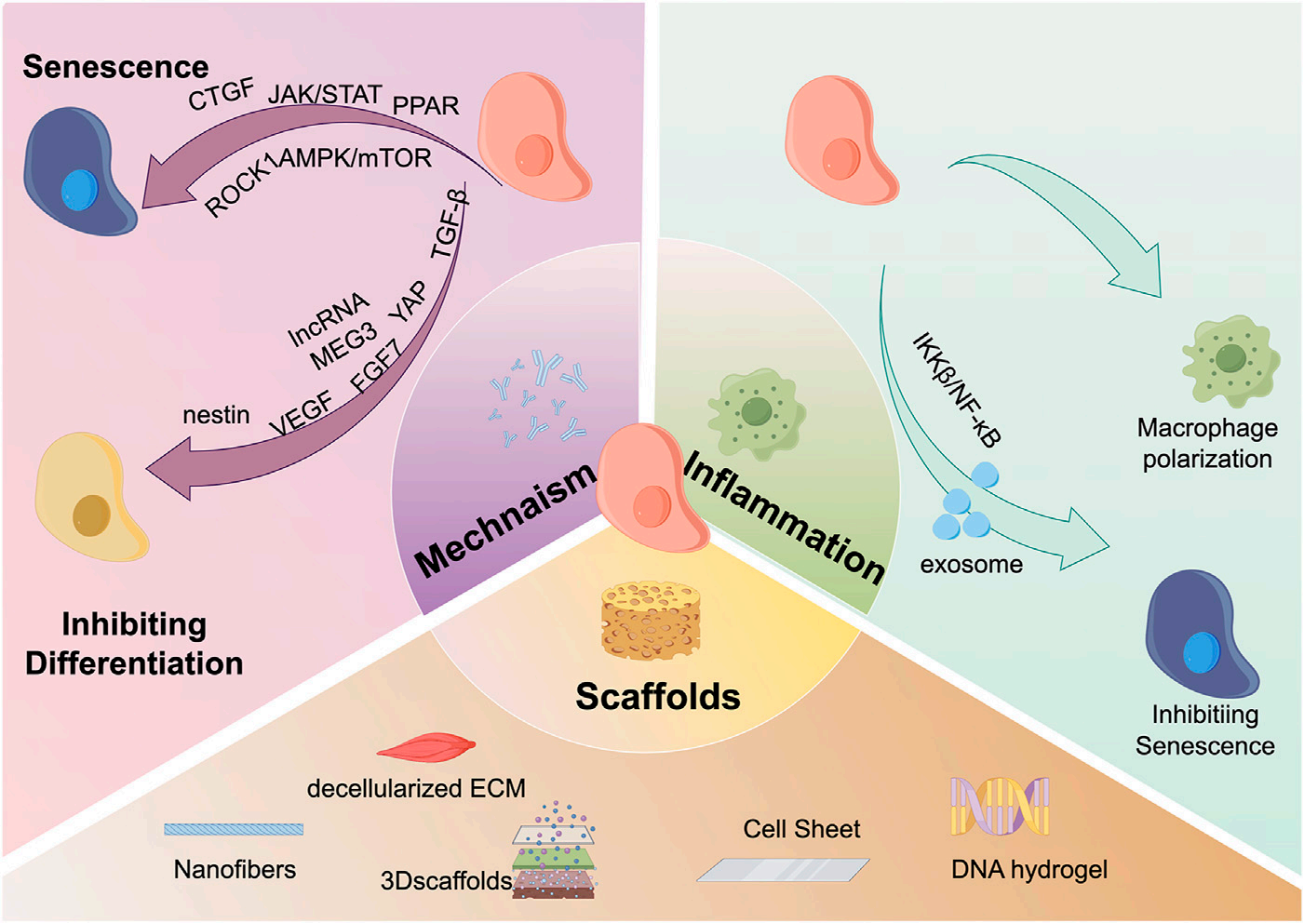
Hot spots and frontiers in the field of TDSCs research in recent years. There are three main research hotspots and frontiers in the field of TDSCs currently. The first is scaffold materials, which mainly include: nanofibers, decellularized ECM, 3D scaffolds, cell sheets, DNA scaffolds, etc.; the second hotspot is inflammation, and TDSCs can directly regulate macrophage polarization. TDSCs can also regulate inflammation-induced cell senescence through the release of exosomes and the IKKβ/NFκB signaling pathway; the third hotspot is the molecular mechanism. TDSCs mainly affects CTGF, JAK/STAT, PPAR, ROCK1, and AMPK./mTOR and other signaling pathways affect cell aging; the cell differentiation ability is regulated through molecular mechanisms such as TGF-β, YAP, FGF7, lncRNA MEG3, VEGF, and nestin.

Keyword analysis is an important bibliometric method for identifying research frontiers. Based on the citation burst of keywords, we can identify the current research frontiers in the TDSCs field. Using the keyword burst function of CiteSpace software, Figure 6D presents the keyword burst of the top 25 TDSCs keywords over the past 20 years. The blue line indicates the continuous appearance of keywords, while the red line indicates intensive appearances. It is evident that the earliest intensively appearing keywords were human bone marrow, marrow stromal cell, fibroblast, and bone. Based on the co-occurrence map of keywords, the current research hotspots have shifted towards scaffolds, mechanisms, and inflammation (Figure 7).

#### 4.2.1 Scaffolds

The two most crucial considerations in tendon tissue engineering were seed cells and biological scaffolds. The topography of the cellular microenvironment plays a vital role in cell activities, guiding cell attachment, morphology, proliferation, and differentiation. Furthermore, it influences cell signaling and morphology Cells can sense morphological changes in the extracellular matrix of cells and convert this information into morphological changes, thereby affecting cell differentiation (Sanie-Jahromi et al., 2023). In 2010, Yin Zi’s team introduced bioengineered scaffolds into the TDSCs research field by planting human-derived TDSCs on electrospun nanofiber scaffolds (Yin et al., 2010). Their findings revealed that aligned electrospun nanofibers were more favorable for TDSCs differentiation towards tendons compared to disorganized scaffolds. Moreover, apart from synthetic scaffolds, natural tissue-derived scaffolds are also employed in various tissue engineering studies (Capella-Monsonis et al., 2023). Three years later, Yin Zi’s team incorporated decellularized biological scaffolds into TDSCs research. By comparing the effects of three decellularized ECMs from tendon tissue, bone tissue, and dermal tissue on seeded TDSCs, they discovered that all three ECMs promoted TDSCs adhesion and proliferation. Notably, the decellularized ECM of bone facilitated TDSCs differentiation towards osteogenesis, while the decellularized ECM of tendon tissue promoted TDSC differentiation towards tendon formation. Based on this characteristic, they studied a unique scaffold composed of tendon decellularized ECM and TDSCs, which, in an *in vivo* study, demonstrated enhanced tendon maturation and biomechanical properties (Yin et al., 2013). Two-dimensional scaffolds fail to replicate the three-dimensional conditions of the extracellular matrix within the body. As an alternative to two-dimensional scaffolds, researchers have explored the application of TDSCs to three-dimensional scaffolds. Compared to two-dimensional scaffolds, three-dimensional scaffolds are more conducive to the tendon differentiation of TDSCs. Sihao Li et al. observed that TDSCs cultured in three-dimensional scaffolds exhibited stronger tenogenic differentiation through the phosphatidylinositol 3-kinase (PI3K)/Akt kinase (AKT) signaling pathway, along with reduced inflammatory phenotypes (Li et al., 2023). *In vivo* studies employing three-dimensional scaffolds revealed a reduction in ectopic calcification complications. Considering the biocompatibility concerns of cell scaffolds, cell sheet technology has rapidly developed Connective tissue growth factor and ascorbic acid can promote TDSCs to secrete extracellular matrix, forming a cell sheet resistant to trypsin breakdown. In addition to TDSCs, the cell sheet contains a substantial number of active substances secreted by TDSCs, including factors inducing tendonogenesis, osteogenesis, and chondrogenesis differentiation (Mifune et al., 2012). Pauline Po Yee Lui et al. (Long et al., 2022) utilized rat TDSCs to culture a cell sheet *in vitro*, wrapping it around tendons for anterior cruciate ligament reconstruction. They observed improved morphology, imaging, biomechanics, and early tendon healing in the cell sheet group (Lui et al., 2014). Early functional exercise post-surgery is crucial for patient functional recovery, indicating that the cell sheet may serve as a valuable graft improvement method. To mitigate immunogenicity issues, which affect post-transplant healing. Removing cellular immunogenicity from the cell sheet may be a better option, indicating that the cell sheet can be applied to large-scale applications rather than personalized customization. In further research, Pauline Po Yee Lui et al. (Shang et al., 2023) chose to decellularize the TDSCs-formed cell sheet, and found that the decellularized cell sheet also promoted graft healing, tunnel bone formation, and angiogenesis (Yao et al., 2023). Hence, the extracellular matrix in the cell sheet may play a pivotal role in promoting healing.

Injecting TDSCs into injured tendon areas has been reported to facilitate injury repair (Gomez-Florit et al., 2022). However, due to tendon tissue sliding, effective attachment of TDSCs to the tendinopathy site is challenging, potentially explaining the limited efficacy of transplanted TDSCs *in vivo* (Gomez-Florit et al., 2022). To address this issue, Guanglin Wang et al. developed a DNA hydrogel scaffold encapsulating TDSCs. This DNA hydrogel scaffold extends the retention time of TDSCs on the tendon, providing an artificial microenvironment conducive to better nutrition, thereby promoting tendon injury repair (Ge et al., 2023).

#### 4.2.2 Mechanism

Tendon aging is a significant contributor to tendon injuries. It leads to structural and functional changes, rendering tendons more susceptible to degeneration and damage. Tendon aging is related to functional changes in TDSCs. TDSCs undergo senescence during the aging process of tendons. Aging TDSCs exhibit reduced proliferation, migration, and multi-lineage differentiation capabilities compared to their younger counterparts. These changes hinder the repair potential of aging tendons. Numerous studies have investigated the mechanisms underlying TDSCs aging. For example, the downregulation of Pin1 and FoxP1 expression has been observed during TDSCs aging (Chen et al., 2015a; Xu and Liu, 2018), affecting peroxisome proliferator-activated receptor (PPAR) (Han et al., 2023), Janus kinase (JAK)/signal transducer and activator of transcription (STAT) (Chen et al., 2021a), protein kinase AMP-activated catalytic subunit alpha (AMPK)/mechanistic target of rapamycin kinase (mTOR) (Dai et al., 2023), non-canonical Wnt signaling pathway (Chen et al., 2021b) and autophagy (Nie et al., 2021a). These pathways are believed to be effective in improving TDSCs aging.

During tendon aging, specific proteins are of interest during tendon aging. Tenomodulin serves as a marker protein for tendon cells and plays a crucial role in TDSCs proliferation, multi-lineage differentiation, and other cellular functions. Research indicates that Tenomodulin is an important gene influencing TDSCs aging. Knocking out Tenomodulin can significantly accelerates TDSCs aging (Alberton et al., 2015). Connective TGF (CTGF) is a cysteine-rich secreted protein expressed widely across various tissues and organs (Yan et al., 2022). Bone morphogenetic proteins (BMP) play a pivotal role in bone development, but their overexpression in aging tendons can result in ectopic calcification (Dai et al., 2020). In aging TDSCs, CTGF expression decreases significantly and correlates with the expression of the aging marker p16. The application of recombinant CTGF protein has been shown to significantly enhance the self-renewal and differentiation capabilities of aging TDSCs (Rui et al., 2019).

Fluid loss is a common occurrence in various tissues during the aging process, and it is closely associated with the function of aquaporins (Kim et al., 2023). YunFeng Rui et al. discovered that aquaporin expression decreased during tendon aging and interfering with its expression can alleviate age-related decline in cell function. The JAK/STAT signaling pathway mediates the impact of aquaporins on aging (Chen et al., 2020). Noncoding RNAs play crucial roles in tendon-related diseases. Chen Lei et al. found that circular RNA (circRNA) PVT1 (circPVT1) regulated TDSCs aging by targeting microRNA (miR)-199a-5p, which further downregulated sirtuin 1 (Han et al., 2022). Additionally, miR-135a inhibits Rho-associated coiled-coil containing protein kinase 1 (ROCK1), thereby improving TDSCs aging (Chen et al., 2015b). Cellular senescence affects the stiffness and size of TDSCs cells, consequently influencing their physical properties and cellular functions (Dulińska-Molak et al., 2014). As cells age, TDSCs stiffness and size decrease; however, inhibiting ROCK1 can restore the stiffness of aging TDSCs (Kiderlen et al., 2019).

TDSCs express numerous markers specific to tendon-related cells, including scleraxis and tenomodulin, and possess the ability to differentiate into tendon tissue. TDSCs play a pivotal role in tendon regeneration and maintenance. Apart from understanding the mechanisms underlying aging, targeting TDSCs differentiation is crucial for maximizing their potential applications. Researchers have discovered that Irisin and activated platelet-rich plasma promote TDSCs differentiation towards tendons, resulting in greater expression of tendon markers and exhibiting potential for tendon treatment (Zhang et al., 2019b; Xu et al., 2022). Weifeng Han et al. reported that p16/miR-217/EGR1 can restore the tenogenic potential of aging TDSCs (Han et al., 2017). Hongwei Ouyang’s team conducted microarray screening on newborn rats and found that the expression of transcription factor Fos decreased over time. Fos is a vital gene in early tendon development and significantly promotes the tenogenic differentiation of TDSCs (Chen et al., 2017). Using single-cell sequencing technology, Hongwei Ouyang’s team observed significant activation of nestin during a specific period of tendon development. Nestin^+^ TDSCs exhibited superior tendon differentiation capabilities compared to nestin^−^ TDSCs, highlighting their importance during tendon development (Yin et al., 2016). In addition to promoting tendon differentiation, inhibiting TDSCs differentiation towards osteogenesis and adipogenesis can reduce tendon complications. Long noncoding RNA MEG3 inhibits the osteogenic differentiation potential of TDSCs through the miR-129-5p/transcription factor 4 (TCF4)/β-Catenin axis, thus reducing ectopic ossification caused by trauma in tendon tissue (Liu et al., 2023b). Vascular endothelial growth factor (VEGF) and PPAR gamma can also suppress adipogenic differentiation of TDSCs, presenting them as promising therapeutic targets for tendon degeneration (Lai et al., 2021; Lai et al., 2022). The recruitment and accumulation of endogenous TDSCs at the site of tendon injury initiate their participation in the repair process. TGF-beta, secreted by TDSCs themselves, is considered an inducing factor for TDSCs recruitment (Tan et al., 2021). Over-activating specific cellular molecular signals or employing recombinant cytokines are commonly used methods to study the repair effects of TDSCs. Periostin (Wang et al., 2021), Yes-associated protein (YAP) signaling pathway (Lu et al., 2023; Wang et al., 2023), mTOR signaling pathway (Nie et al., 2021b), and Fibroblast growth factor 7 (FGF7) (Zhang et al., 2022) have been reported to enhance cellular functions of TDSCs, thereby promoting tendon repair.

#### 4.2.3 Inflammation

Inflammation plays a pivotal role in the context of tendon injuries, particularly regarding the involvement of TDSCs. Following an injury, the body initiates an inflammatory response as part of the natural healing process. This inflammation not only aids in clearing damaged tissue but also regulates the activation and behavior of TDSCs. Inflammatory factors released by inflammatory cells during this process can reduce collagen secretion, leading to vasodilation and decreased blood vessel density, which subsequently affects TDSCs. In the presence of inflammation, TDSCs become activated, contributing to the repair and regeneration of injured tendon tissue. Activated TDSCs improve the inflammatory response in tendon tissue, increase the secretion of IL10, inhibit the polarization of macrophages towards M1 (Tarafder et al., 2017), and promote the M2 polarization of macrophages (Mao et al., 2022). Annexin A1 and CD200 serves as an important marker and drug target in tendon inflammatory diseases (Liu et al., 2018; Giancola et al., 2022). Excessive inflammation may induce senescence in TDSCs. Studies have found that nonsteroidal anti-inflammatory drugs can alleviate inflammation-induced TDSCs aging and promote the progression of tendon degeneration (Cai et al., 2022). I-kappaB kinase beta (IKKβ)/nuclear factor of kappa light polypeptide gene enhancer in B cells (NF-κB) is activated in degenerated tendon tissue, and targeted inhibition of IKKβ/NF-κB improves inflammation-induced TDSCs senescence. Furthermore, in conditions of diabetes, the differentiation ability of TDSCs decreases, which is closely related to inflammatory cytokines. Du-Hwan Kim et al. found that using Migration Inhibitory Factor can directly enhance the differentiation ability of TDSCs under high blood sugar conditions (Kim et al., 2021). Exosomes, small membrane-enclosed sacs involved in cell-to-cell communication and molecule transportation within the body, have gained significant attention in the fields of biology and medicine (He et al., 2018; Kalluri and LeBleu, 2020). They play a crucial role in various physiological and pathological processes, including inflammation-related diseases (Noonin and Thongboonkerd, 2021), and TDSCs-derived exosomes have shown therapeutic effects on tendon inflammation and apoptosis after Achilles tendon injury (Zhang et al., 2020). Additionally, drug monomers like tectorigenin have been found to improve the inflammatory response of TDSCs through the inflammatory signaling pathway NF-κB and MAPK (Moqbel et al., 2020). Aspirin is a classic anti-inflammatory drug. Wang (Wang et al., 2019) reported that aspirin can reverse the inflammatory response induced by IL-1β in TDSCs.

### 4.3 Strengths and limitations

This study possessed several strengths. Firstly, it corrected and updated similar articles in the original publication. Although an earlier article has employed bibliometrics to study TDSCs, their data was not filtered and it included numerous irrelevant papers. Consequently, the results failed to accurately represent the current status and hotspots in the field of TDSCs research. Our study provided accurate literature data that truly reflected the correct research status. Secondly, we conducted a comprehensive literature review guided by research hotspots. Thirdly, we summarize the research focus, cell sources, intervention factors, experimental methods, experimental indicators and main research findings of the current original research on TDSCs, and provide a detailed summary table for scientific researchers to find. However, our study only included data from one database, potentially resulting in the omission of some relevant research.

## 5 Conclusion

Tendon stem cells are crucial seed cells for tendon tissue engineering and hold significant research prospects. Currently, scaffolds, molecular mechanisms, and inflammation regulation have been prominent research areas in this field.

## Data Availability

The raw data supporting the conclusion of this article will be made available by the authors, without undue reservation.
