# Supplementary material for: Global research trends and hotspots on tendon-derived stem cell: a bibliometric visualization study
Source: Front Bioeng Biotechnol. 2024 Jan 8;11:1327027. doi: 10.3389/fbioe.2023.1327027 (PMC10801434; doi:10.3389/fbioe.2023.1327027)
Supplement: Supplementary file 1 [file DataSheet1.PDF]

Supplementary Table 1: Summary of TDSCs-related studies

Focus	Source	Culture Methods	Stimulus Method	Assessment Methods	Evaluation Index	Main Study Findings	Paper	DOI
Cell Properties	Mice	LG-DMEM supplemented with 10% FBS, 2 mM GlutaMax, 5 μg ml^{-1} gentamicin	Collagenase used to digest tissue	Centrifugation and plating for MSC isolation	MSC migration and adhesion	Cells from both tendon and bone were capable of migrating from the tissue and adhering to the plate, indicating the presence of MSCs.	Campbell 2019	10.1016/j.jse.2019.02.008
	Mice	LG-DMEM with FBS, penicillin, and streptomycin.	Cell density gradient	Cell morphology and colony formation assay<br><br>Cell growth<br><br>Trilineage differentiation experiment<br><br>Immunocytochemical staining<br><br>Flow cytometry analysis	Cell proliferation ability, senescence rate, Trilineage differentiation<br><br>Potential, stem cell marker	a pure population of TSPCs could not be isolated from mice digital flexor tendons through culturing cells at a density gradient. Cells seeded at low densities had very limited proliferative ability and did not show more prominent stem cell characteristics when compared with cells seeded at high densities.	Wu 2020	10.1089/scd.2020.0036
	Horse	HG-DMEM with FBS and penicillin-streptomycin.	Differentiation media for adipogenesis, osteogenesis, and chondrogenesis; growth factors like BMP-12 and	Cell proliferation assays, tenogenesis gel assays, histology, and image analysis for cell alignment and gel contraction.	Expression of genes related to adipogenesis, osteogenesis, chondrogenesis, and tenogenesis. Cell alignment and	Identified distinct tendon stem cell phenotypes with varying differentiation potentials and proliferative capacities.	Rajpar 2020	10.1186/s13287-020-01640-8

			IGF-1 for tenogenesis.		contraction in gels.			
	Rat	DMEM with 20% FBS and antibiotics	Nanotopographic cues provided by NOA86 and PUA substrates with different stiffness levels.	qRT-PCR for gene expression, H&E staining for histological examination, and immunofluorescence staining for cell identification.	Expression of type I and III collagen, and gene expression of scleraxis.	The differentiation of TDSCs is affected by the mechanical stiffness and nanotopography of the culture substrate, which has implications for tendon regeneration and healing.	Kim 2018	10.2147/IJN.S181743
	Mice	DMEM with 10% FBS and 1% penicillin/streptomycin	None	qPCR	Gene expression profiling (e.g., Scleraxis, Tenomodulin, Collagen 1α2, Elastin, TGFβ2)	Identification of distinct gene expression profiles dependent on treatment, developmental stage, and anatomical origin of TPCs	Brown 2014	10.1016/j.jbiomech.2013.09.018
	Mice	DMEM with 10% FBS and 1% penicillin/streptomycin	None	Cell proliferation assay, qPCR	Gene expression levels (e.g., Scx, Tnmd, Col I, Eln, TGFβ2)	Analysis of gene expression in mesenchymal stem cells (MSCs) and TPCs, revealing insights into tenogenic potential and differentiation pathways	Brown 2015	10.1186/s13287-015-0043-z
	Horse	HG-DMEM supplemented with 20% fetal bovine serum, 37.5 μg/ mL of ascorbic acid, 300 μg of L-	Preplating	Monolayer expansion, cell proliferation, Flow Cytometry, Immunophenotyping, qPCR	tenogenic genes, scleraxis (Scx) and tenomodulin (Tnmd)	Preplating and subsequent monolayer expansion did not alter the immunophenotype ($CD29^{+}$, $CD44^{+}$,	Durgam 2016	10.1089/ten.TEC.2016.0152

		glutamine/mL 100 U of sodium penicillin/mL, and 100 µg of streptomycin				CD90^{+}, and CD45^{-}) and trilineage differentiation capacity of TDPC fractions		
	Shark	Not given	None	Histological analysis and in situ hybridization	MyHC, Scx	one of the HCs gives rise to tendon progenitor cells of the EOMs, which is an exceptional condition in our previous understanding of head muscles	Kuroda 2021	10.1186/s40851-021-00170-2
	Rat	DMEM with 10% FBS and 100mg/ml streptomycin and 100U/ml penicilin	Mechanical tension: 1HZ frequency and 8% elongation	Osteogenic differentiation assays RT-PCR, WB	RUNX2,GAPDH, Wnt5a, Wnt5b, Ror2 Rac1,pJNK,JNK,	UMT induced the osteogenic differentiation of rTDSCs via Wnt5a/Wnt5b/JNK signal pathway.	Liu 2015	10.1159/000430117
	Mice	LG-DMEM that was supplemented with 10% fetal bovine serum, 100 U/ml penicillin, and 100 mg/ml streptomycin	Mechanical stretch: cyclic uniaxial stretching with 1 Hz at 4% and CFTR kncodown	WB, RT-PCR, Sirius red staining，Immunoprecipitation assay，Immunofluorescence and immunohistochemistry，Ultrasound imaging	Scx, Mkx, Col1A1, Tnmd, Dcn, Bgn, CFTR et al.	CFTR plays an important role in tenogenic differentiation and tendon regeneration by inhibiting the b-catinin/pERK1/2 signaling pathway.	Liu 2017	10.1096/fj.201601181R
	Human	HG-DMEM with 10% FBS and 50 U/mL penicillin, 50	Different growth factor treated	IF, RT-PCR	DCN,SCX, MKX, TN-C, COL1A1	TGF-β3 is the main inducer of scleraxis, an early expressed tendon marker, while at the same time inhibiting tendon	Orfei 2018	10.3390/ijms2001

		mg/mL streptomycin, 2 mM L-glutamine				markers normally expressed later, such as decorin.		0149
	Human	LG-DMEM with 100 IU/mL penicillin, 100 mg/mL streptomycin and 10% FBS	None	RT-PCR, differentiation potienal,	Osteogenic, adipogenic, tendon differentiation,ALP, PPAR, LPL,COL-I/II, MMP-1/2,N-CAD,Cx43	human rotator cuff tendon stem cells and human long head of the biceps tendon stem cells can be isolated and possess a high regenerative potential, which is comparable with that of BMSCs. Moreover, comparative analysis of the sphingolipid pattern of isolated cells with that of BMSCs and fibroblasts revealed the possibility of using this class of lipids as new possible markers of the cell differentiation status.	Randelli 2013	10.1177/0363546512473572
	Rat	DMEM with 100 IU/mL penicillin, 100 mg/mL streptomycin and 10% FBS and 2 mM L-glutamine	mechanical load :0.5 Hz at 4% or 8%	WB, RT-PCR, ALP Activity Assay, Alizarin Red S Staining	BMP-2 and Osteogenic differentiation	Activation of BMP-2 expression in TDSCs during tendon overuse might provide a possible explanation of ectopic calcification in calcifying tendinopathy	Rui 2010	10.1002/jor.21218
	Rat	LG-DMEM 100 IU/mL penicillin, 100 mg/mL streptomycin and 10% FBS and 2 mM L-glutamine	BMP-2 treatment	Alizarin red S staining ,ALP activity assay , WB, RT-PCR	BMPR and Osteogenic differentiation	both TDSCs and BMSCs exhibited stem cell properties, including clonogenicity and multi-differentiation potential. TDSCs expressed higher mRNA and protein levels of BMP receptors IA, IB and II. They also exhibited higher	Rui 2011	10.1007/s00264-011-1417-1

						osteogenic differentiation with and without BMP-2 stimulation compared with BMSCs.		
	Rat	LG-DMEM 100 IU/mL penicillin, 100 mg/mL streptomycin and 10% FBS and 2 mM L-glutamine	Tendon surgery	Colony-forming, BrdU, Fluorescence-activated cell sorting, b-gal, Osteogenic and Chondrogenic differentiation, IHC	Cell viability, cell aging, Osteogenic and Chondrogenic ability, Col1a1, Scx, Tnmd	TDSCs (CI) showed altered fate, a higher cellular senescence, but a lower proliferative capacity compared to TDSCs (HT), which might contribute to pathological chondro-ossification and failed tendon healing in this animal model.	Rui 2013	10.1089/scd.2012.0555
	Human	a-MEM supplemented with 20 % FBS and 1 % penicillin and streptomycin and 100 mM 2-mercaptoethanol	None	Colony-forming assay<br>Multi-potent differentiation<br>RT-PCR and Multi-potent differentiation	TNMD, CD146, Osteogenic, Adipogenic and Chondrogenic ability,	The age-related variations in human TSCs affect the number of isolated cells and their self- renewal potential, while multi-potency assays are not influenced by tendon ageing, even though cells from younger individuals expressed higher levels of osteogenic and adipogenic genes, while chondrogenic genes were highly expressed in cells from older individuals.	Ruzzini 2013	10.1007/s00167-013-2457-4
	Human	DMEM/Ham’s f12 (1:1) with 10% FBS and 1% penicil-lin/streptomycin	None	PCR,	Scx, TNMD, ALP, Col1A2, RUNX2, LPL, PPAR, COL2a1,FOS, VIM, PTGS2, EF1A	LHB tendon might be a suitable cell source for regenerative approaches, both in inflamed and non-inflamed states.	Schmalzl 2019	10.1302/2046-3758.89.

	Rat	DMEM containing 10% FBS, 50 mg/ml ascorbic acid	different concentration of dexamethasone	Colony formation assay, Collagen Accumulation	Collagen, colony formation number	dexamethasone causes a concentration-dependent inhibition of TDC number and collagen accumula- tion.	Scutt 2005	10.1002/jor.20030
	Rat	DMEM, 10% FBS 100 U/ml penicillin, 100 mg/ml streptomycin	MECHANICAL TENSILE：<br><br>0.5 Hz sinusoidal curve, 2% elongation	ALP assay,ACTIVE RhoA DETECTION,RT-PCR, WB	ALP, RhoA, Runx2, GAPDH, Wnt5a, beta-actin	UMT induced osteogenic differentiation of rTDSCs via the Wnt5a-RhoA pathway, which might contribute to ectopic ossification in tendon tissue due to mechanical loading.	Shi 2012	10.1002/jcb.24190
	Rat	LG-DMEM containing 10% FBS, 100 U/mL penicillin, 100 mg/mL streptomycin, and 2 mM l-glutamine	Vitro Passaging	Colony-forming assay, BrdU, Fluorescence ,b-gal assay, Multidifferentiation Potentials, RT-PCR	b-actin, C/EBPa PPAR, Col2A1 Acan, Tnmd, Scx, Alpl, Bglap	As tendon stem cells are passaged in vitro, stem cell properties decrease	Tan 2012	10.1089/scd.2011.0160
	Human	DMEM with 10% FBS	None	IHC, WB, In situ hybridization, RT-PCR	CD133, GAPDH, HPRT, SMA, Msi-1, Scx, Nestin, Col-I, Col-III, Smad8, CD29, CD44	the perivascular niche may be considered a source for tendon precursor cells.	Tempfer 2009	10.1007/s00418-009-0581-5
	Rat	Not given	TNC overexpression	IHC, wounder healing assay, RNA-sequencing, WB	Migration ability, MMP-9, p-FAK, FAK, p-AKT, AKT, integrin,	TNC regulated the migration of STSCs via ITGA9, thereby promoting the regeneration of tendon injuries	Xu 2021	10.1002/biof.1759

	bovine	LG DMEM with 15% FBS 100 U/ml penicillin, 100 mg/ml streptomycin, 2.5 ng/ ml bFGF, and 2 mM L- glutamine	None	Colony formation assay, Multipotency analysis, RT-PCR	Proliferation ability, Multipotency, Collagen I, Collagen II, Collagen III, Tenascin-C, CD44, GAPDH, LPL, PPARr, Sox9, OPN, Runx2, ACAN, ALP	fetal bovine TDSCs not only had strong self-renewal capacity but also possess the potential for multi-lineage differentiation.	Yang 2016	10.1007/s11626-016-0043-z
	porcine	LG-DMEM with 15% FBS 100 U/ ml penicillin, 100 mg/ml streptomycin, 2 mM L-glutamine, 0.4 ng/ml EgF, 2.5 ng/ml bFgF and 2.5 ng/ml SCF	None	Colony formation assay, Multipotency analysis, RT-PCR	Proliferation ability, Multipotency ,Collagen I, Collagen II, Collagen III, GAPDH, LPL, PPARr, SOX9, OPN, Runx2, ALP, ACAN	TDSCs isolated from porcine tendon exhibit the charac- teristics of multipotent stem cells.	Yang 2019	10.3892/ijmm.2018.3545
	Horse	HG-DMEM with110 μg/mL sodium pyruvate, 10% Cellect Silver FBS, 10% horse serum and 100 U/mL sodium	Comparison between TDSC, BMSC and ADSC	Cell cycle assay, RT-PCR, Microscopic imaging, Mechanical testing	GAPDH, SCX, TNMD, COL-I, COL-II, DCN, BGN, ELN, COMP, MHC-I, MHC-II,	TDSCs are the ideal cell type for regenerative medicine therapies for tendinopathies, exhibiting the most mature tendon-like phenotype in vitro.	Youngstrom 2016	10.3109/03008207.2015.1117458

		penicillin, and 100 µg/mL streptomycin sulfate						
	Rabbit	DMEM with 20% FBS	cyclic stretching of 4% or 8% at 0.5 Hz	RT-PCR	COL-I, COL-II, PPAR, Sox9, Runx2	low mechanical stretching may be beneficial to tendons by enabling differentiation of TSCs into tenocytes to maintain tendon homeostasis	Zhang 2009	10.1002/jor.21046
	Rabbit	DMEM with 20% FBS and 100 µM 2-mercaptoethanol 100 U/ml penicillin and 100 µg/ml streptomycin.	None	RT-PCR, Multipotency analysis, IHC, histological	COL-I, COL-II, Sox9, Runx2, GAPDH, PPAR, Multipotency,OCT4 ,SSEA-4	TSCs exhibit distinct properties compared to tenocytes, including differences in cell marker expression, proliferative and differentiation potential, and cell morphology in culture.	Zhang 2010	10.1186/1471-2474-11-10
	mice	α-MEM containing 10% FBS and antibiotic–antimicotic solution	None	RT-PCR, Alkaline phosphatase assay, MTT, North blotting, animal experiments, Histological and immunohistochemical	Cell viability, Scx, Six-1, EphA4, COMP, Col-1, Sox9, Col-X， Col-II, OPN, OC, ALP, Osterix, aP2, PPAR, GAPDH,	the established tendon cell line possesses mesenchymal stem cell-like properties, suggesting the existence of mesenchymal stem cell in tendon tissue.	Salingcarnboriboon 2003	10.1016/S0014-4827(03)00107-1
	Mice	α-MEM containing 10% FBS	Ctsk-CKO	FACS analysis, Colony formation assay and in vitro multipotent differentiation, TUNEL.	CD24, CD44, CD200, CD105, Sca1, Multipotent differentiation toward Osteogenesis,	Identified a subpopulation of TDPCs labeled by Ctsk-Cre, and demonstrated that activation of Hh signaling drives HO in tendons and ligaments in a cell-autonomous manner.	Feng 2020	10.1172/JCI132518

					Adipogenesis, and Chondrogenesis, ALP activity, apoptosis testing.			
	Mice	DMEM supplemented with 20% fetal bovine serum, 100 µM 2-mercaptoethanol, 100 U/ml penicillin and 100 µg/ml streptomycin.	Aging	Immunocytochemical staining, qRT-PCR.	Oct-4, NS, Sca-1, SSEA-1, Nanog, collagen type I, tenomodulin, LPL, Sox-9, Runx-2.	While aging impairs the proliferative ability of TSCs and reduces their stemness, moderate exercise can mitigate the deleterious effects of aging on TSCs.	Zhang 2015	10.1371/journal.pone.0130454
	Mice	DMEM containing 10% FBS and 5% penicillin/streptomycin.	Neonatal tendon progenitor cells (TPC).	Adenovirus-mediated cell labeling.	RFP+/DAPI+ and RosaT+/DAPI+ cells.	Neonatal TPCs improved and restored functional gait by reducing overall scar formation, improving enthesis collagen alignment and altering bony composition response after supraspinatus tendon repair.	Vervaecke 2022	10.1016/j.jse.2022.05.004
	Rat	DMEM with 10% FBS, and 1% penicillin and streptomycin	Young or Aged ECM	Cell proliferation assay, β-gal assay, RT-PCR	SSEA-1, Oct-4, Tnmd, Scx, Cell viability, Senescence cell	The impaired capacity of aged TSCs can be rejuvenated by exposure to young DECM	Jiang 2018	10.17219/acem/75503
	Porcine	DMEM+20%FBS	None	Scanning electron microscopy, Immunostaining, Cell proliferation measurement	CD105, CD31, CD73 and CD146, collagen IV, tubulin	The isolated cells from paratenon and IFM also harbored abundant stem/progenitor cells as evidenced by their ability to form colonies and express	Zhang 2020	10.1007/s00441-020-

					polymerization promoting protein (TPPP), neurotransmitter substance P (SP)	stem cell markers including CD73 and CD146.		03379-3
	mice	HG-DMEM 10%FBS ascorbic acid (50 mg/ml)	None	RNA isolation and RT-PCR<br>Microarray analyses<br>Single-cell qRT-PCR<br>Histological examination<br>Mechanical testing<br>Transmission electron microscopy	CD34, CD18, CD44, CD90, CD105, CD146, Scx, Mkx, Eln, Col I, and Col XIV	these findings provide new insights into the identification of subpopulations of TSPCs and illustrate the crucial roles of nestin in TSPC fate decisions and phenotype maintenance	Yin 2016	10.1126/sciadv.1600874
	Mice	DMEM10%FBS L-Ascorbic-Acid-2-Phosphate MEM-Amino Acid	Tenomodulin	Immunofluorescence<br>PCR, Adipogenic, osteogenic and chondrogenic differentiation assays<br>Colony unit forming (CFU) assay<br>WST-1 assay, ß-galactosidase assay<br>Detection of p16, p21 and P53 on tendon tissue sections, Transfection of mTSPC	CD146, CD105, CD90.2, CD73, CD44, Sca-1, Nestin and Nanog<br>Eya1 and Six1, collagen type I (Col Iα2)	Loss of Tnmd affects significantly the self-renewal and senescence properties but not the multipotential of TSPC.	Paolo 2014	10.1089/scd.2014.0314
	Human	None	aging	AFM, Two-photon excited fluorescence (TPEF) microscopy	Stiffness, ROCK 1 and 2.	cellular stiffness is a suitable marker for cell aging and ROCK a potential target for therapeutic applications of cell rejuvenation.	Stefanie 2018	10.1016/j.bbrc.2019.01.027
	Rat	DMEM 10%FBS	None	Self-renewal and proliferative potential Cell proliferation assay,	FACSCAN	The successful isolation of tendon-derived stem cells under the optimized	Rui 2009	10.1089/ten

		100 U=mL penicillin, 100 mg=mL streptomycin, and 2 mM L glutamine		Immunocytochemical staining, Osteogenic differentiation assays. Adipogenic differentiation assays Chondrogenic differentiation assays Histological assay Immunohistochemical staining RT-PCR	Program，CD34， CD31，CD44， CD90	growth and differentiation conditions was useful for future stem-cell-based tissue regenerative studies as well as studies on their roles in tendon physiology, healing, and disorders using the rat model.		.tea.2009.0529
	Human	DMEM/Ham's F-12 (1:1 mixture) medium supplemented with stable glutamine (365.3 mg/L), 1 × MEM amino acids, 10% FBS and 1% L-ascorbic acid-2-phosphate	None	IHC, PCR, Wounder healing, Cell Sheet Histology, TEM,	Tenogenic-Related Genes, CD146, Nestin, STRO1, histology	hTSPCs exceed hMSC-Scx cells in several characteristics, namely clonogenicity, multipotentiality, gene expression profile and rates of tendon-like sheet formation, whilst in three-dimensional cell sheets, both cell types have comparable in vitro healing potential and collagenous composition of their three-dimensional cell sheets, making both cell types a suitable cell source for tendon tissue engineering and healing.	Hsieh 2018	10.3390/ijms19082272
	murine	DMEM supplemented with 20% foetal calf serum, 100 U/ml penicillin, 100 μg/ml streptomycin and 2 μg/ml amphotericin B	None	Cell proliferation assay, Colony formation assay, Tri-lineage differentiation assays, RT-PCR,	Cell viability, GAPDH, CD90, CD73, TNC, SCX, MKX, Sca-1, Nanog, Tnmd, THSB4, CD45, Runx2, OC, OSX, OPN, FABP4,	The differences in morphology, clonogenicity, stem cell marker expression and multipotency observed between tenocytes and TDSCs indicate that at least two cell populations are present in murine tail tendon.	Lee 2018	10.1186/s12891-018-2038-2

					Leptin, Sox9, Col2A1, AGG			
Disease Mechanism	Rat	LG-DMEM with 10% FBS and antibiotics.	Mechanical load	Microarray analysis, real-time PCR, ALP staining, siRNA interference, and pharmacological inhibition studies	miR-337-3p expression, osteogenic and chondrogenic gene expression	Mechanical loading enhanced chondro-osteogenic differentiation in TDSCs, regulated by miR-337-3p. IRS1/ERK1/2 and Nox4/JNK pathways mediate this process.	Geng 2019	10.1089/scd.2020.0036
	Rat	LG-DMEM with FBS, penicillin, streptomycin, and L-glutamine.	streptozotocin (65 mg/kg)	qRT-PCR assay for gene expression, ALP staining for osteogenic activity, and Safranin O staining for chondrogenic activity.	Expression levels of ALP, BMP2, OPN, OCN, Col II, SOX9, Col I, TNMD, Scx.	Diabetic TDSCs showed higher osteogenic and chondrogenic differentiation potential but lower tenogenic potential compared to healthy TDSCs.	Shi 2019	10.1186/s13287-018-1108-
	Rat	DMEM with FBS and antibiotics, expanded after reaching confluence.	Cholesterol at various concentrations	Cell counting kit-8 assay for proliferation, wound healing assay for migration, cell cycle and apoptosis assays, TUNEL assay for DNA fragmentation, western blotting for protein analysis.	Cell proliferation, migration rate, cell cycle phase distribution, apoptotic cell percentage, autophagic flux.	High cholesterol affects cell viability by inhibiting proliferation, inducing apoptosis, and affecting cell cycle progression. It also induces autophagy and apoptosis through ROS-activated pathways in tendon-derived stem cells.	Li 2020	10.1186/s13287-020-01643-5
	Rat	DMEM with 10% FBS	Alteration of mechanical and biological environment leading to osteogenic differentiation	Histopathological examination, immunofluorescence staining, gene expression analysis.	Presence of chondrocyte and osteoblast markers, differentiation potential of TDSCs.	Erroneous differentiation of TDSCs contributes to the pathogenesis of calcifying tendinopathy. Re-direction of TDSCs differentiation as a potential treatment.	Rui et al.	No DOI (PMID: 21362289)

			potential.					
	Rat	LG-DMEM with 10% FBS	Interleukin-10 (IL-10) treatment	Cell proliferation assay, cell cycle analysis, wound healing assay, RT-qPCR, western blot analysis, immunofluorescence staining	Cell proliferation, migration, gene expression of tendon cell markers (e.g., Scx, Col1), protein expression levels	IL-10 enhances cell proliferation and migration but inhibits tenogenic differentiation in TDSCs in vitro. IL-10 activates the JAK/Stat3 signaling pathway, which seems to be involved in the regulation of these processes	Deng 2018	10.3892/mmr.2018.9547
	Mice	DMEM with 20% FBS	Tendon surgery	Colony-forming capacity, cell surface marker expression, multipotency assays, transplantation into injured Achilles tendons	Tenogenic and chondrogenic differentiation, ectopic endochondral ossification	TPCs from injured tendons exhibit strong chondrogenic potential. CD105-negative subpopulation shows superior chondrogenic potential and is associated with chondroid degeneration in injured tendons	Asai 2014	10.1002/stem.1847
	Mice	TDSCs in MED with 10% FBS.	Botox-induced tendon unloading	Histological Analysis, CMP Staining, Polarisation Microscopy, Immunofluorescence, qPCR, Western Blot, Statistical Analysis	Tendon morphology, TDSC viability and differentiation capacity	Botox injection impairs TDSC growth, viability, and differentiation potential, contributing to tendon atrophy	Chen 2021	10.1186/s13287-020-02084-w
	Rat	DMEM with 10% FBS and 100 U/ml penicillin, 100 mg/ml	PEG-2 or BMP-2	Alizarin red staining<br>Alkaline phosphatase staining<br>WB<br>PCR	AKT, pAKT, pERK1/2, ERK1/2 pSMAD1,5,8, BMP-2	the PI3K-Akt signaling cascade is essential for PGE2- induced BMP-2 production and BMP-2-mediated osteogenic differentiation, suggesting that PI3-kinase- Akt signaling contributes to	Liu 2013	10.1016/j.bbrc.2012.11.083

		streptomycin, and 2 mM L-gluta- mine				the formation of calcified tissues in tendinopathy.		
	Rat	LG-DMEM with 10% FBS and 50 µg/ml penicillin, 50 µg/ml streptomycin 100 µg/ml neomycin	Collagenase induce tendon injury	PCR, WB, ICC	BMP-2, BMP-4, BMP-7, BMPRIA, BMPRIB, BMPRII, beta-actin, SMAD1/5/8, pSMAD1/5/8	BMPs and the BMP/Smad signaling pathway paly an important rol in the pathogenesis of tendinopathy.	Lui 2013	10.1186/1471-2474-14-248
	Rat	DMEM supplemented with 10% FBS, 100 units/mL penicillin, and 100 µg/mL streptomycin	Nesfatin-1	Multipotency analysis, CCK-8, IF, wounder healing, IHC, histological, Animal experiment, RT-PCR, WB	Multipotency, cell viability, migration ability, Human GAPDH, Human NUCB2, Rat GAPDH, Rat Scx, Rat Mkx, Rat Tnmd, Rat ALP, Rat Col1a1, Rat RUNX2,CD29, CD44,CD45, CD90, Col1A2, RUNX2, LC3B/A, P62, GAPDH, ATG5	nesfatin-1, associated with diabetes mellitus promotes the osteogenic differentiation of TDSC and the pathogenesis of HO in rat tendons via the mTOR pathway	Xu 2020	10.3389/fcell.2020.547342

	Human	DMEM with 20% FBS	PGE2	cell proliferation, Multipotency analysis, Immunostaining	SSEA-4, BMP-2, osteogenic differentiation	BMP-2 mediates PGE2-induced reduction of proliferation and osteogenic differentiation of hTSCs.	Zhang 2011	10.1002/jor.21485
	Human, Mice	L-DMEM, 10% fetal bovine serum, and 1% penicillin-streptomycin.	Different tissue: High-quality transcriptome data from 372 single-cell samples, including 195 samples from patient 1 and 177 samples from patient 2.	H&E staining, immunofluorescence staining, Assessment of Multi-Differentiation Capacity, Flow Cytometry Analysis and Sorting, scRNA-Seq,	MT2A, CSF3, CXCL5, CXCL8, CXCL3, CD34, PCNA, BMI1, SOX2, CD74, TNNT1, MYOD1, MYF5, CHRNA1, ACTC1, MYF6, PAX7, TNNT3, THBS4, POSTN, BGN, COL1A1, FMOD, COMP, PRG4, LHFP,	The research identified four main subtypes, including stem cell, muscle, tendon, and muscle-tendon progenitor cells (MTP). The MTP subpopulation, which remains the characteristics of stem cells and also expresses muscle and tendon marker genes simultaneously, may have the potential for bidirectional differentiation. The research also found the muscle-tendon progenitor cells were distributed in the shape of a transparent goblet; muscle cells first connect to the MTP and then to the tendon.	Yan 2022	10.34133/2022/9760390
	Rat	DMEM containing 20% fetal bovine serum, 100 U/ml penicillin, 100 µg/ml streptomycin, and 2 µM glutamine.	Aging	Quartz thickness shear mode (TSM) resonators.	the mechanical properties of tendon stem cells (TSCs), The admittance spectrums of TSM with TSC monolayer.	It was shown that aging TSCs were large, flat and heterogeneous in morphologies while young TSCs were uniformly elongated. Increased cell size and irregular cell shape might be associated with the dense cytoskeleton organization, which could lead to an increase in both stiffness and viscosity.	Wu 2015	10.1016/j.snb.2014.12.117

	Human	DMEM/Ham's F-12 With 10% FBS and 1% L-ascorbic acid-2-phosphate	Aging	Flow cytometry (FACS) and immunocytochemistry Self-renewal analysis and WST-1 Genome-wide microarray PCR, Scratches,Western blotting and ELISA	Col 1, FN, ROCK, lnt α5, lnt αV, lnt β1, lnt β3, lnt β5	This study provides the first fundamental basis for further exploration into the molecular mechanisms behind tendon aging and degeneration as well as for the selection of novel tendon-specific therapeutical targets.	Julia 2013	10.1111/acel.12124
	Rat	high glucose DMEM 10%FBS	Hyperuricemia	CCK-8, RT-qPCR, WB, Biomechanical Testing	phosphoAKT, AKT, phosphomTOR, mTOR, phosphop70S6K,p70S6K,phospho-4E-BP1,4E-BP1,p62,LC3A/B,β-actin	asymptomatic hyperuricemia may be a predisposition of ATR by impeding the normal functions of TSPCs. This information may provide theoretical and experimental basis for exploring the early prevention and care of ATR.	Jingjing 2022	10.1155/2022/6795573
	Human	α-MEM, 10% FBS, 100 U P/S, and 250 ng/mL amphotericin	Injury time	TDSCs Proliferation Capability Assay, RT-qPCR, Trilineage Differentiation Assay, Four-point scoring (FPS)system, total degeneration score, hematoxylin and eosin (H&E) staining and Saffron O and Fast Green staining, transmission electron microscope	Tendon and fibrocartilage regeneration markers: TGF-β1, aggrecan, COL2A1, and COL3A1	Differentiation ability of TDSCs derived from the rotator cuff remnant was reduced with age and chronicity. Histological degeneration of remnant tendon deteriorated with chronicity. Remnant in the greater tuberosity was still alive, but those in young or acute injury	Huang 2021	10.1016/j.arthro.2021.09.027
	Rat	low-glucose Dulbecco's modified Eagle medium, 10% fetal	Injury	Animal experiments, Histology and immunofluorescent staining	Genes expression , IdU, Oct4, Nanog, Sox2, nucleostemin, CD146	The pluripotency markers and pericyte-related marker in LRCs might be important for function after injury.	Tan 2013	10.1089/scd.2013.0073

		bovine serum, 50 mg/mL penicillin, 50 mg/mL streptomycin, and 100 mg/mL neomycin						
	Mice	alpha-MEM, 2 mM L-glutamine, antibiotics/antimycotics, 100 µm 2-mercaptoethanol, 20% FBS	Different region	Animal experiments, Conditioned media experiment, RT-qPCR	Genes: Scx, Tnmd, Emcn, Bgn, Dcn, Col1a1, Col3a1, Col5a1, Col11a1, Col12a1, Col14a1	These findings highlight the synergistic potential of including these progenitor populations in restorative tendon engineering strategies.	Mienaltowski 2014	10.1186/scrt475
	Rat	LG-DMEM supplemented with 10% FBS	Microstructural disruption	CCK-8, RT-PCR, WB, IF, Chondrogenic differentiation assay,	Cell viability, CD44, CD45, CD90, Col1, SCX, Tnmd, Lpl, PPAR, Col2, Sox9, Runx2, ALP, Aggrecan, AFT-4, beta-actin	The study indicated that tendon microdamage could induce the chondrogenic differentiation of TDSCs through triggering ER stress to activate ATF-4 and SOX9 subsequently.	Liu 2023	10.1002/jor.25362
	Rat	LG-DMEM with 10% FBS 100 U/mL penicillin, 100mg/mL streptomycin	D-Glucose	MTT, Apoptosis assay, RT-PCR, WB	Cell viability, apoptosis, beta-actin, Scx, Col1a1, Tnmd, Col1,	These findings might account for some pathological mechanisms underlying the pathogenesis of diabetic tendon disorders.	Lin 2017	10.18632/oncotarget.15418
	Mice	LG-DMEM with 15 FBS % and 1%	Achilles tenotomy model	Animal experiment, Histologic, Histochemical and IHC, Micro-CT,	SHH, IHH, SMO, GLI1, OCN,	Hh signalling contributes to trauma-induced tendon ossification and affects	Li 2022	10.3390/ant

		penicillin/streptomycin		RT-PCR, ALP Staining, Alcian Blue Staining,	RUNX2, AGG, COL2A1,	ROS generation through antioxidant pathway in osteogenic differentiation of TDSCs, indicating that targeting Hh signalling by GANT58 may be a potential treatment for trauma-induced tendon ossification.		iox11112265
	Rat	(DMEM) with 20% FBS and 1% penicillin/streptomycin	chemically-induced tendinopathy or injury-induced tendinopathy	Animal experiment, RT-PCR, WB, Fluorescence-activated cell sorting (FACS) analysis, Immunocytochemical staining, Multipotency,	Oct4, SSEA4, Tnmd, CD18, CD45, CD90, cell viability, multiple lineage differentiation, ColI, ColII	TDSCs from injury-induced tendinopathy showed markedly high proliferation and high expression of type III collagen and α-SMA compared to other groups. Adipogenic potentials in TDSCs from injury-induced tendinopathy were also higher.	Kim 2018	10.1002/jcp.26475
Treatment target or drug treatment effect	Rat	DMEM with 10% FBS, 100 U/mL penicillin, 100 mg/mL streptomycin	rCTGF	Western Blot, CCK-8 Assay, Colony-Forming Ability (CFA) Assays, Cell Cycle Analysis by flow cytometry.	Scx, Tnmd, nestin, Col1a1 (tendon-related markers).	CTGF plays a vital role in TSPC aging, CTGF treatment leads to increased expression of tendon-related markers and decreased senescence.	Rui 2019	10.1155/2019/6257537
	Rat	DMEM with 10% FBS, 100 U/ml penicillin, 100 mg/ml streptomycin, 2 mM L-glutamine	Aspirin (0, 0.25, 0.5, 1, 2, 5 mM); Wnt3a protein and GSK-3β inhibitor or LiCl; COX-2 inhibitor NS398.	Hoechst 33342 staining, Annexin V-FITC Apoptosis Detection Kit, Western blotting, Immunostaining.	Apoptosis markers: Bcl2, cleaved caspase-3, P-β-catenin, COX-2.	Aspirin induces apoptosis in rat TSCs via the mitochondrial/caspase-3 pathway and inhibits the Wnt/β-catenin pathway. Elevated COX-2 levels may protect cells against apoptosis.	Wang 2018	10.1159/000495050

	Mice	DMEM with 10% FBS, 100 U/ml penicillin, 100 mg/ml streptomycin, and 2 mM L-glutamine,	Knockdown of lncRNA KCNQ1OT1 with siRNA.	qRT-PCR and Western blot to assess gene expression; RNA immunoprecipitation (RIP) and RNA pull-down assays.	Adipogenic and osteogenic differentiation markers: PPARγ, RUNX2, Adiponectin, Osterix.	Knockdown of lncRNA KCNQ1OT1 suppresses adipogenic and osteogenic differentiation of TSCs via miR-138.	Yu 2018	10.1080/1538410 1.2018.1534510
	Rat	DMEM with 10% FBS	BGN (50, 100, or 500 ng/ml).	Cell viability and proliferation assay; Western blot; qRT-PCR.	Tendon-related protein levels of THBS-4 and TNMD increased; expression of chondrogenic and osteogenic markers like SOX9, ACN, and RUNX2 decreased.	BGN enhances tenogenic differentiation and suppresses osteogenic/chondrogenic differentiation via BMP7/Smad1/5/8 pathway.	Zhang 2019	10.1002/jcp.28247
	Human	LG-DMEM with 10% FBS and 1% PSG	Ferulic acid (FA) at different concentrations (0, 5, 10, 15 μM).	Colony formation assay, CCK-8 kit for cell proliferation, multi-differentiation assay, qRT-PCR, Western blot.	Differentiation markers: PPARγ, Col2A1, Acan, Runx2, EGR1; stemness markers: CD44, CD73, CD90, CD105.	FA improves self-renewal and multi-differentiation potential of hTSCs, induces hypoxia and upregulates EGR1 expression, HIF1α and EGR1 are crucial for FA's enhancing effects on hTSCs.	Qiu 2019	10.1002/dvg.23291
	Rat	DMEM with 10% FBS, 1%	Hepatocyte growth factor	Proliferation assay (CCK-8, Ki67 detection), scratch assay, Transwell	Phosphorylation levels of ERK1/2,	HGF promotes TDSC proliferation and migration but inhibits osteogenic	Han 2019	10.1002/jcp

		antibiotics,	(HGF) treatment	assay, mineralization assay, immunofluorescence, Western blot.	AKT, Smad1/5/8, expression of osteogenic markers (Alp, Runx2, Col1A1).	differentiation; signaling pathways (PI3K/AKT, MAPK/ERK1/2, HGF/c-Met) modulate these effects.		.2836 0
	Rat	LG-DMEM containing 20% FBS and 100 mM 2-mercaptoethanol.	Cholesterol treatment at concentrations of 0, 1, 10, or 100 mg/dL.	Quantitative polymerase chain reaction (qPCR), Western blot analysis, Measurement of intracellular ROS production.	Gene expression levels of Scx, Tnmd, Col1, Col3, Lum, Egr1, Fmod; Protein levels of Scx, Tnmd, Col1, Col3, CAT, NOX4, IκBα, p-IκBα, p-65, p-p65; ROS levels using peroxide-sensitive dye and flow cytometry.	High cholesterol inhibits tendon-related gene expressions in TDSCs through ROS-activated NF-κB signaling, suggesting a potential mechanism for tendinopathy in hypercholesterolemia.	Li 2019	10.10 02/jcp .2843 3
	Rat	DMEM with 10% FBS	Aspirin (0–2 mM) and GDFs (GDF6, GDF7, GDF11) for various days.	RNA-seq, Western blot, qRT-PCR, immunostaining, biomechanical testing.	Tenogenic markers (TNC, TNMD, SCX), expression of GDF7, GDF11, and P-Smad1/5.	Aspirin enhances tenogenic differentiation and improves healing in tendinopathy. GDF7 facilitates differentiation via Smad1/5 signaling pathway.	Wang 2020	10.10 02/jcp .2935 5
	Rat	DMEM with 20% FBS and antibiotics.	Nicotine(10,100,1 000ng/ml) or ascorbic acid(5, 50, 500ng/ml)	CCK-8, Wounder Healing, qRT-PCR, WB, apoptosis assay	Cell proliferation, migration ability, apoptosis rate, TNMD, SCX,	Addition of ascorbic acid partially reversed the inhibitory effect of a high concentra- tion of nicotine.	Shi 2019	10.10 80/03 00820 7.201

					Collagen I, PPAR, Runx2, Sox9			9.1663349
	Rat	DMEM with FBS, penicillin, streptomycin, and L-glutamine.	Exosome treatment	Western blotting; Immunostaining, Tri-lineage differentiation assay, Histomorphometry biomechanical analysis of tendons	Expression of collagen type I, tenomodulin, MMP-3, TIMP-3; mechanical properties of tendons like maximum loading and stress.	Tendon-derived exosomes promote tendon healing by balancing ECM synthesis and degradation, enhancing tenogenesis of TSCs.	Wang 2019	10.1111/jcmm.14430
	Rat	DMEM with FBS and penicillin-streptomycin.	Exosome treatment.	EdU assay for cell proliferation, scratch and transwell assays for migration, western blotting for protein analysis.	Proliferation rate, migration ability, protein expression levels related to proliferation and migration pathways.	TSC-Exos promoted tenocyte proliferation and migration in a dose-dependent manner, potentially through PI3K/AKT and MAPK/ERK1/2 signaling pathways.	Zhang et al.	10.1186/s13287-020-01918-x
	Rat	DMEM with FBS and antibiotics,	AGA as a Runx2 inhibitor and T0070907 as a PPARγ inhibitor were used. Optimal doses were determined via a cytotoxicity	Osteogenic and adipogenic differentiation was assessed using specific media and staining methods. qRT-PCR and Western Blot analysis were performed.	Expression levels of Runx2, PPARγ, collagen type I, collagen type III, tenomodulin.	AGA blocked osteogenic differentiation and promoted tenogenic differentiation at mRNA level. T0070907 inhibited adipogenic differentiation without affecting tenogenic differentiation.	Kim et al.	10.3390/ijms21082687

			assay.						
	Rat	DMEM with FBS and antibiotics.	VH298 treatment in various concentrations.	Cell viability assay (CCK-8), Western blotting, scratch test, real-time PCR.	Cell proliferation, migration, gene expression of collagen-1a, collagen-3a, decorin, tenomodulin, tenascin C, Sox-9, aggrecan, col-2a.	VH298 enhanced TDSC functions, promoting proliferation, migration, and chondrogenic differentiation, influencing healing in a rat model.	Qiu 2018	10.1016/j.bbrc.2018.09.172
	Rat	DMEM with 20% FBS and 100 mM mercaptoethanol	PRP and TDSC	Immunohistochemistry, Real-time PCR, Western Blots	mRNA and protein expression of collagen I, collagen III, tenascin C, and Smad 8	Synergistic effects on tendon healing when combining TSCs and Platelet-Rich Plasma (PRP) under both loaded and unloaded conditions	Chen 2011	10.1002/jor.22033
	Rat	DMEM with 20% FBS	Mechanical stretching and Platelet-rich clot releasate (PRCR) treatment	Western Blotting, Flow Cytometry, ELISA	Protein concentrations of collagen types I and III, cell differentiation markers (PPARγ, SOX-9, RUNX2), growth factors (TGF-β1, VEGF)	PRCR stimulates proliferation and suppresses differentiation of TSCs towards non-tenocyte lineages which might impede tendon healing	Chen 2012	10.1177/1473230012040 00418
	Rat	DMEM with 10% FBS, penicillin,	Dexamethasone treatment	Flow Cytometry, Cell Proliferation Assay, qPCR, Western Blotting,	Scleraxis gene expression,	Dexamethasone inhibits the differentiation of TSCs into tenocytes by	Chen 2015	10.1016/j.js

		streptomycin, and L-glutamine		ChIP-PCR Assay, Immunostaining, Immunohistochemical Staining	tenocyte marker expression	downregulating scleraxis gene expression		bmb.2015.04.010
	Rat	DMEM with 20% FBS	IL-6 treatment	Cell Proliferation Assay, Cell Cycle Analysis, qPCR, Immunoblot Analysis	Cell proliferation, cell cycle phases, gene and protein expression levels	IL-6 promotes proliferation but inhibits tenogenic differentiation of TDSCs via the JAK/STAT3 pathway	Chen 2018	10.12659/MSM.908802
	Horse	HG-DMEM supplemented with 10% fetal bovine serum, 37.5μg/mL of ascorbic acid, 300 μg of L-glutamine/mL 100 U of sodium penicillin/mL, and 100μg of streptomycin sulfate/mL	0, 0.007, 0.07, and 0.7nM insulin	MTT,Osteogenic Differentiation<br>Alizarin Red Staining<br>Alkaline Phosphatase Bioactivity Measurements<br>RT- PCR	Runx2，Alkaline phosphatase，Osteonectin，Insulin receptor IGF-I receptor ,EF1-alpha	hyperinsulinemia may alter TPC phenotype and subsequently impact the quality of repair tendon tissue.	Durgam 2019	10.1155/2019/1602751
	Human	The medium consisted of 10% fetal bovine serum penicillin streptomycin 90% DMEM	FGF-2	PCR,WB,Histological assessments, Biomechanical testing	FGF2, Collagen-III, SCXA, GAPDH	hTDSCs modified with the FGF2 promote and improve the quality of tendon repair compared with that of hTDSCs alone.	Guo 2019	10.1016/j.bbrc.2019.10.082

	Rat	DMEM plus 10% FBS, 1% penicillin–streptomycin, and 2mM L-glutamine at 37°C in 5% CO_2	CHIP overexpression	RT-PCR, Immunofluorescence staining assay, MTT, WB, Ectopic implantation and histological examination	CHIP<br>Tenomodulin (Tnmd)<br>Scleraxis (Scx)<br>Type I collagen (Col I)<br>Type III collagen (Col III)<br>Decorin<br>Runt-related transcription factor 2 (Runx2)<br>Alkaline phosphatase (ALP)<br>Osteocalcin<br>Peroxisome proliferator-activated receptor-γ (PPARγ) Adipocyte protein 2 (AP2)<br>Adiponectin<br>β-Actin	CHIP is an important contributory factor to tenogenic tissue formation.	Han 2017	10.1093/abbs/gmx005

	Rat	Not given	IGF-1, TGF-beta1, GDF-5	Multipotency studies<br>Immunohistochemistry analysis<br>ELISA<br>Histological staining	CD31, CD34, CD44, CD90, DCN, SCX, osteonectin, collagen types I and II, FABP4 and TEN-C	GDF-5 promotes the transition of tendon stem cells towards tenocytes; TGFβ1 induces differentiation along several pathways, including a phenotype indicative of fibrocartilage or calcified tendon, common problems in tendon healing; and IGF-1 promotes proliferation and maintenance of TSC phenotypes, thereby creating a population sufficient to have a beneficial effect	Holladay 2014	10.1002/term.1852
	Human	DMEM containing 20% FBS, 37°C 5% CO2	BMAC–PRP complex	Immunocytochemical Staining<br>Fluorescence-Activated Cell Sorting (FACS) Analysis<br>Multidifferentiation Potential<br>Immunohistochemical Staining<br>Migration Assay<br>CCK-8<br>Evaluation of Shoulder Function	Type I and Type III Collagens<br>VAS and ASEs scores	BMAC–PRP enhances the proliferation and migration of TDSCs and prevents the aberrant chondrogenic and osteogenic differentiation of TDSCs, which might provide a mechanistic basis for the therapeutic benefits of BMAC–PRP for rotator cuff tendon tear	Kim 2017	10.3727/096368917X694705
	Rat	DMEM) containing 10% fetal bovine serum (FBS), 1%	Exosome treatment	CCK-8,Wound healing, WB,IHC	CD64, Alix, pERK1/2, TGF-beta, p-SMAD2/3, SMAD2/3,beta-actin	TSC-exo is a po- tential strategy for treating tendon injuries	Li 2021	10.1016/j.bbrc.2020.12.057

		penicillin, and streptomycin.						
	Rat	α-MEM with 10% FBS and 100 units/ml streptomycin + 100 units/ml penicillin	Tectorigenin Or TNF-α	Viability， Apoptosis Assay RT-PCR,WB,， ALP, Alizarin Red,Senescence Assessment ,IF,IHC	MMP-3 MMP-9 MMP-13 COX-2 iNOS Col1 Runx-2 Scx ,Mkx Tnmd IL-6 IL-10 18S,pP38,P38, JNK,p-JNK,ERK, p-ERK, p65, IKBα，p- IKBα	Tectorigenin treatment decreases activation of NF-kappa B and MAPK signaling in TDSCs. Tectorigenin ameliorates tendinopathy in the in vivo rat model.	Moqbel 2020	10.3389/fcell.2020.568894
	Rat	DMEM with 20% FBS 100 U/mL penicillin, and 100 mg/mL streptomycin	Pure platelet-rich plasma or leukocyte-rich PRP	population doubling time assay, Immunosorbent Assay RT-PCR, Multidifferentiation Potentials	Cell proliferation, Collagen, growth factor detemination,Nanog , Oct-4, Col-1, Tenascin C, PPRA, Sox9, Runx2, mPGES, IL-1beta, GAPDH	L-PRP produced lower levels of growth factors .TSC proliferation was significantly decreased in L-PRP in a concentration-dependent manner.	Zhang 2016	10.1177/0363546516644718
	Rabbit	DMEM with 10% FBS and 100 U/mL penicillin, 100 mg/mL	EGR1 overexpress	IF, RT-PCR, WB, rotator cuff injury model, histological and IHC	EGR1, BMP12, p-SMAD1/5/8, Smad1, SCX, TNMD, TNC,	EGR1 plays a key role in tendon formation, healing, and repair through BMP12/Smad1/5/8 pathway.	Tao 2015	10.1159/000369730

		streptomycin, and 2 mM l-glutamine			COL1, beta-actin			
	Human	LG-DMEM with 10% FBS and 1% penicillin-streptomycin	MiR124	luciferase assays, RT-PCR, WB, Sirius red staining	GAPDH, COLA1, COLA2, TNC, DCN, FN1, EGR1, miR124-3p	miR124 suppressed collagen for- mation during the tendon differentiation of hTDSC while anti-miR124 promoted it. Furthermore, egr1 knockdown abolished the promotive effect of anti-miR124, suggesting that miR124 prevents tendon differentiation via suppressing egr1 expression. Therefore, miR124 may be a promising therapeutic target for tendon injury.	Wang 2016	10.1016/j.yexcr.2016.08.018
	Rat	DMEM with 10% FBS	Aspirin	RNA-seq, wb, RT-PCR, Biomechanical testing	PTEN, p-PI3K, PI3K, p-AKT, AKT, GAPDH, PPAR,	down-regulating PTEN/PI3K/AKT signalling, aspirin inhibited adipogenesis of TSCs and fatty infiltration in injury tendon, promoted biomechanical properties and de- creased rupture risk of injury tendon	Wang 2019	10.1111/jcmm.14622
	Rat	LG-DMEM with 10% FBS and 100 U/mL penicillin, 100 mg/mL streptomycin	Platelet-rich plasma	Multi-differentiation assays，,IF, Cell viability, Cell cycle analysis, Cell migration assay, Capillary-like tube formation, RT-PCR, Histology, Biomechanical testing	Collagen I, Collagen III, Tenascin-C, Scleraxis, GADPH	PRP can activate TDSCs to improve the quality of Achilles tendon rupture healing in the early stages.	Xu 2020	10.1002/term.2020
	Rat	DMEM supplemented with	Pioglitazone	Multipotency analysis	Multipotency, cell viability, apoptosis,	Pio reversed exacerbated osteogenic capacity induced by AGEs both in vitro	Xu 2020	10.1111/jc

		10% FBS and 100 units/mL penicillin, and 100 µg/mL streptomycin		CCK-8, Apoptosis analysis,WB, IF, Animal experiment, Histological analysis	LC3, P62, C-Cas3/9, P53, P21,	and in vivo, which provides a potential therapeutic target for heterotopic ossification prevention.		mm.14901
	Rat	DMEM with 20% FBS	celecoxib	Cell proliferation assay, Multipotency analysis, RT-PCR, WB	Scx, Mkx, Egr1, Col1a1, Col3a1, Col5a1, Col6a1, Col14a1, Tnmd, Bgn, Dcn, Fmod, Lum, Tnc, Fn1, Fbn1, Eln, GAPDH,	celecoxib inhibits tenocytic differentiation of tendon-derived stem cells but has no effects on cell proliferation.	Zhang 2014	10.1016/j.bbrc.2014.06.058
	Rat	LG-DMEM with 10% FBS and 1% penicillin–streptomycin	CTGF treatment	Histology and immunohistochemistry RT-PCR	histological assay results and Col-1, IL-6, IL-10, MMP-3, TIMP-3	anti-inflammatory roles of CTGF-stimulated TSCs that are likely associated with improved tendon healing	Tarafder 2017	10.1096/fj.201700071R
	Human	DMEM with 10% FBS and 1% penicillin-streptomycin	PAR1-PRP or PAR4-PRP	IF, RT-PCR, histological assay	histological assay ,Collagen I, Collagen II, MMP-1, MMP-2, LPL, Runx-2, GAPDH	Selective activation of PRP using PAR1 and PAR4 has dif- ferential effects on differentiation, proliferation, and gene expression of TSCs as well as on wound healing of injured tendons, which is due to differential release of pro-and anti-angiogenic factors, VEGF and endostatin, respec- tively.	Zhang 2019	10.1177/2041731418820034
	Rat	DMEM supplemented with 10% FBS and 100	Irisin	CCK-8, Colony formation assay, WB, RT-PCR，IF, ELISA, Proteasome activity assay	proliferation, bFGF, C-MYC, CTGF, Col1, Scx, Tnmd,	irisin and agents targeting YAP/TAZ may be promising therapeutic options for tendinopathy.	Xu 2022	10.1007/s11626-

		units/ml penicillin and 100 µg/ml streptomycin			Yap, 18S, FNDC5, GAPDH, TAZ			022-00699-2
	Mice	None	Tamoxifen	Patellar Tendon Injury, Histology and Immunohistochemistry, Two Photon Imaging.	SMA9, ScxGFP, GDF5-9, SHG, anti-tenascin-C, tdTomato.	SMA9+ cells in the tendon midsubstance are an amplifying progenitor population during growth. These cells do not contribute to the fibrocartilage within the tendon enthesis and ligamentous cells within the knee, which originate from a Gdf5 lineage.	Dyment 2014	10.1371/journal.pone.0096113
	Rat	LG-DMEM, fetal bovine serum and penicillin plus streptomycin.	Conditioned medium (CM) of tendon stem cells (TSCs) induced with hepatocyte growth factor (HGF).	Flow cytometry analysis, Proteome Profiler Array and Bioinformatic Analysis, CCK-8 Assay, Wound Healing Assay, Western Blotting	CD90, CD44 (cell surface marker cluster), cell viability, cell migration rate, COL III, fibronectin, MMP-1, MMP-9.	HGF stimulates the secretion of soluble secretory products by TSCs and CM promotes the repair and functional recovery of ruptured Achilles tendon. Thus, HGF-induced TSC CM has therapeutic potential for the treatment of tendinopathy.	Zhang 2021	10.3389/fcell.2021.654084
	Human	DMEM supplemented with 20% fetal bovine serum, 100 U/ml penicillin, and 100 µg/ml streptomycin.	Dexamethasone (Dex)	digital cellometer, qRT-PCR.	proliferation effects, differentiation effects, collagen type I, PPARγ, Sox-9, Runx-2.	Dex treatment in clinics may cause a paradoxical effect on the injured tendons it is supposed to treat: by inducing non-tenocyte differentiation of hTSCs, Dex treatment depletes the stem cell pool and leads to the formation of non-tendinous tissues (e.g., fatty and cartilage-like tissues), which make tendon susceptible to rupture.	Zhang 2012	10.1002/jor.22193

	Rat	DMEM containing 10% fetal bovine serum and 1% penicillin-streptomycin antibiotic mixture	Hepatocyte growth factor (HGF)–expressing tendon stem cells (TSCs)	Flow cytometry, Oil Red O, Alizarin Red, Alcian Blue, Lentiviral Transfection, Western Blotting.	CD44, CD90, CD11b, CD106, Adipogenic potential, Osteogenic potential, Chondrogenic potential, COX-2, COLIII, α-SMA.	TSCs + HGF, which exhibit HGF overexpression, may promoting tendon healing via decreasing inflammation and fibrosis, perhaps partly via inhibiting TGF-β1-induced signaling.	Zhang 2021	10.3389/fcell.2021.659389
	Human	α-MEM supplemented with 2 mM L-glutamine, 1% antibiotic-antimycotic mixture, and 20% FBS	Gangliosides	Osteogenic and Adipogenic Differentiation, Metabolic Radiolabeling of Cell Sphingolipids, Extraction and Chromatographic Separation of Radiolabeled Sphingolipids, Real-Time PCR, Analysis of Mineralization, Immunoblotting.	ALP, PPAR-γ, LPL, GM1 synthase, Osteogenic Differentiation, p-PDGFR-β, PDGFR-β.	Ganglioside GM1 significantly increases during osteogenic differentiation of hTSCs. Most importantly, the ganglioside increase is instrumental for driving the process through the inhibition of PDGFR-β.	Bergante 2018	10.1155/2018/4706943
	Human	DMEM supplemented with 20% fetal bovine serum, 100 μM 2-mercaptoethanol, 100 U/ml penicillin and 100 μg/ml streptomycin.	Oxygen	Cell Proliferation Experiment, Immunocytochemistry, Multi-differentiation Potentials, qRT-PCR.	NS, Oct-4, SSEA-4, Adipogenesis, Chondrogenesis, Osteogenesis, Collagen type I, Tenascin C.	Oxygen tension is a niche factor that regulates the stemness of hTSCs, and that less oxygen is better for maintaining hTSCs in culture and expanding them for cell therapy of tendon injuries.	Zhang 2013	10.1371/journal.pone.0061424

	Mice	DMEM supplemented with 20% fetal bovine serum and 1% penicillin and streptomycin.	Running	Immunocytochemistry, Sircol collagen assay.	Nucleostemin, Cellular Collagen Production.	Exercise exerts its anabolic effects on tendons at least in part by increasing proliferation to expand the pool of TSCs and also by increasing TSC-related cellular production of collagen, the predominant component of tendons.	Zhang 2010	10.1002/jor.21123
	Human	DMEM plus 20% FBS.	Prostaglandin E2 (PGE2)	Immunocytochemical analysis,	Oct-4, Nanog, NS,	PGE2 can exhibit biphasic effects on hTSCs, indicating that while high PGE2 concentrations may be detrimental to tendons, low levels of PGE2 may play a vital role in the maintenance of tendon homeostasis in vivo.	Zhang 2014	10.1371/journal.pone.0087706
	Rat	DMEM with 10% fetal bovine serum, 100U/mL penicillin, 100 mg/mL streptomycin and 2 mM L-glutamine.	overexpressing and RNA interference CTGF TSCs.	ELISA, CTGF shRNA and transfection, qRT-PCR, Immunofluorescence staining, Western blot, Chemical crosslinking assay.	ColI, CTGF, Scx, Tnmd, Tn-C, BMPR1a, BMPR1b, Runx2, Sox9, PPARγ	BMP12 stimulated expression of tenocyte differentiation markers and that CTGF promoted this effect via the Smad1/5/8 signaling pathway.	Liu 2015	10.1159/000373994
	Rabbits	DMEM supplemented with 20% fetal bovine serum and 1% penicillin and streptomycin.	Platelet-rich plasma (PRP)	Cell size and shape, RT-PCR, Immunocytochemistry, Immunocytochemistry, Western blot, Sircol collagen assay kit, Population doubling time (PDT), qRT-PCR.	Collagen types I and III, Total collagen, Cell proliferation rate, Nucleostemin, α-SMA, Collagen.	The PRCR treatment also markedly enhanced TSC proliferation, tenocyte-related gene and protein expression, and total collagen production, all of which indicated that PRCR treatment induced differentiation of TSCs into activated tenocytes.	Zhang 2010	10.1177/0363546510376750

	Mice, Rabbits	DMEM supplemented with 20% fetal bovine serum, 100 µM 2-mercaptoethanol, 100 U/ml penicillin and 100 µg/ml streptomycin.	Running, PGE2.	Enzyme immunosorbent assay, Oil Red O Assay, Alizarin Red S Assay.	PGE2, Adipogenesis Osteogenesis.	After a bout of rigorous treadmill running, mice tendons produced higher levels of PGE2 than tendons of cage control mice, and PGE2 decreased TSC proliferation and induced adipogenic and osteogenic differentiation of TSCs in an apparent dose-dependent fashion.	Zhang 2010	10.1002/jor.20962
	Human, Rat	LG-DMEM, 10% heated inactivated fetal bovine serum, 50 µg/mL penicillin, 50 µg/mL streptomycin, and 100 µg/mL neomycin.	Different doses of vitamin C or hydrogen peroxide.	Colony-Forming Assay, Cell Migration Assay, Alamar Blue and BrdU Assays, Caspase Assay.	CFU, Cell migration assay, Cell viability, Proliferation, Apoptosis.	Oxidative stress affects both recruitment and survival of tendon progenitor cells, while antioxidants may exert beneficial effects at low doses.	Lee 2017	10.1155/2017/8785042
	Mice	DMEM containing 20% FBS.	IL-1β	Proliferation activity, Tenogenic differentiation, Tenogenic, Chondrogenic, Osteogenic, Adipogenic Differentiation, Qpcr, Immunoblot analysis, Lactate measurement.	DNA contents, Scx, Mkx, Egr1, Col3, Col1, Tnmd, Bgn, Dcn, Fmod, Lum, MMP13, Agg, SOX9, Osx, Runx2, Cebpa, Cebpg, HKII, LDHA, PDHA.	IL-1β strongly and irreversibly impairs tenogenic potential and alters glucose metabolism in tendon progenitors appearing in injured tendons. Inhibition of IL-1β may be beneficial for maintaining function of tendon progenitor cells during the tendon repair process.	Zhang 2015	10.1016/j.bbrc.2015.05.122

	Rat	DMEM with 10% fetal bovine serum, 100 U/ml penicillin, 100 mg/ml streptomycin, and 2 mM L-glutamine.	sirtuin (Sirt)1	RT-QPCR, Western blot, Alizarin red staining, Oil Red O staining.	Sirt1, Runx2, PPARγ, BMP2.	Sirt1 promotes the osteogenic differentiation of TSCs through upregulating β-catenin and Runx2 and inhibits the adipogenic differentiation of TSCs through the PI3K/AKT pathway with downregulation of CEBPα and PPARγ.	Liu 2016	10.3892/mmr.2016.5417
	Human, Mice	DMEM containing 10% FBS and 1% antibiotics-antimycotics.	CTRP3	Stained with Alcian blue (pH 1.0) and Nuclear Fast Red solution, Alizarin Red S, Sphere formation assay, qRT-PCR	Chondrogenic differentiation, Adipogenesis, Osteogenesis	CTRP3 elicited a transcriptomic pattern that stimulates abnormal differentiation of tendon stem/progenitor cells and ectopic chondrification as an effect linked to activation of Akt signaling.	Cho 2021	10.1126/sciadv.abg6069
	Human, Rat	MSC expansion media	retinoic acid receptor (RAR), Scx	qRT-PCR, Immunocytochemistry, Differentiation assays, Western blots	SCX, Oct4, Aggrecan, CD1530, CD2665, TGFβ2, all-trans retinoic acid, BIX-01294, C646, Adipogenesis, Osteogenesis, and Chondrogenesis Differentiation	The effect of RAR compounds on TSCs is reversible by revealing their multi-lineage differentiation ability upon withdrawal of the compound.	Webb 2016	10.1186/s13287-016-0306-3
	Rabbits	Ham's F12: high glucose DMEM 1:1; supplemented with 10% fetal bovine	Mechanical loading	The effect of mechanical stretch on cell proliferation and matrix synthesis in BMSC/TC co-culture, RT-PCR.	CollagenI, Alkaline Phosphatase, Osteopontin,	Proliferation and differentiation of local precursor cells could be enhanced by mechanical stimulation, which results in	Song 2017	10.1159/000460005

		serum and 1% penicillin/streptomycin.			Tenascin C and Tenomodulin.	enhanced regenerative potential of BMSCs and TCs in tendon-bone healing.		
	Rat	DMEM supplemented with 20% fetal bovine serum, 100-U/mL penicillin, and 100-mg/mL streptomycin.	Simvastatin	Co-culture assay, ALP, Alizarin red, and Von Kossa staining, Safranine, Alcian blue, and toluidine blue staining, Immunofluorescence staining, RT-Qpcr.	Chondroblast, Osteogenesis differentiation, VEGF, COL1A1.	Local administration of simvastatin can promote the tendon-bone healing by enhancing neovascularization, chondrogenesis, and osteogenesis in different stages of the tendon-bone healing process.	Ni 2022	10.1007/s00441-022-03714-w
	Human	α-MEM supplemented with 2 mM glutamine, 1% antibiotic-antimycotic mixture , and 20% fetal bovine serum.	Lipogems Product Treatment	Cell Morphology and Proliferation, Flow cytometry, Adipogenic Differentiation, Osteogenic Differentiation, qRT-PCR, Wound-Healing Assay.	Cell viability, Cell Apoptosis, CD9, CD73, HLA-DR, CD13, CD29, CD44, CD45, CD71, CD90, CD105, CD106, CD34, CD166, HLA-ABC, NG2PE, SSEA-4, Lineage Cocktail , CD18, CD140a, CD140b, CD146, Stro-1, CD117, COL1A1, TNMD, Nanog, Oct4,	The Lipogems product significantly increases the proliferation rate of hTSCs without altering their stemness and differentiation capability. Moreover, treated cells increase the expression of VEGF, which is crucial for the neovascularization of the tissue during the healing process.	Randelli 2016	10.1155/2016/4373410

					KLF4, VEGF, PPAR-γ, LPL, ALP, MYOG, MYOD, S14, Cell Migration			
	Rat	DMEM containing 10% fetal bovine serum, 100 U/ml penicillin, 100 mg/ml streptomycin and 2 mM L-glutamine.	Dexamethasone (Dex)	qRT-PCR, Western blotting, Immunostaining, Oil red staining and HE staining.	Scleraxis, C/EBPα, aP2, DKK1, aP2, C/EBPα, DKK1, Phospho-GSK-3α/β (ser21/9), Phospho-GSK-3α/β (tyr279/tyr216),	By upregulating DKK1 expression, reducing the level of P-GSK-3β (ser9), and increasing the level of P-GSK-3β (tyr216), Dex causes the degradation of β-catenin, the central molecule of the classical WNT pathway, thereby inducing rat TSCs to differentiate into adipocytes.	Chen 2015	10.1159/0004385 38
	Rat	DMEM with 10% FBS	Eriocitrin	CCK8, wound-healing assay, RT-PCR,	Cell viability ,apoptotic , migration ,COMP, Fibronectin, and Biglycan	eriocitrin promoted cell proliferation of tendon stem cells, improved the migration of tendon stem cells, and inhibited the expression levels of some scar formation-related gene markers.	Shang 2021	10.1134/S16076729 21050045
	Human	DMEM with 10% FBS and 2 mM L-glutamine	Circular RNA PVT1	β-gal staining, CCK-8 assay, wounder healing, WB, Luciferase assay, WB, RT-PCR	Self renewal, migration, and tenogenic differentiation, P16, Scx, Tnmd, Col1a1	circPVT1 is a novel potential therapeutic target for reducing tendon senescence.	Han 2021	10.1016/j.tiv.2021.1052 97
	Human	α-MEM with 2 mM glutamine,1% antibiotic-	hypoxia	WB, trypan blue dye exclusion assay, MTT Assay, RT-PCR	HIF-1α Lamin A/C	These results support the notion that hypoxia, by activating HIF, plays a crucial role in preserving stem cells in an	Alessandra 2018	10.1155/2018/946 8085

		antimycotic mixture,20% FBS				undifferentiated state in the “hypoxic niches” present in the tissue in which they reside before migrating in more oxygenated areas to heal a damaged tissue.		
	Human	LG-DMEM with 10% FBS and 1% penicillin-streptomycin solution	rapamycin, N-acetylcysteine (NAC), 3-methyladenine (3-MA), or chloroquine (CQ)	Colony formation assay, Cell proliferation assay, qRT-PCR, Differentiation assays, WB shRNA transfection	Nanog，Oct-4, NS, and SSEA-4, and impaired differentiation capability.	Thus, the findings of this study suggest that autophagy prevents oxidative stress-induced loss of self-renewal capacity and stemness in hTSCs through suppression of ROS accumulation.	Hua 2016	10.1159/000447916
	Rat	LG-DMEM with 10% FBS and 1% penicillin-streptomycin solution	IGF-1，BMP-2	RNA interference, Oil Red O Staining, qRT-PCR, WB	adipogenic differentiation, p-PKA, PKA, CEBP, HDAC and b-actin	IGF-1 and BMP-2 together mediate PGE2-induced adipogenic differentiation of TSCs in vitro via a CREB- and Smad-dependent mechanism.	Junpeng 2014	10.1371/journal.pone.0085469
	Human	DMEM+10%FBS+ 1% penicillin/streptomycin	hypoxia	RT-qPCR, Alcian blue staining assay, Western blot, Immunohistochemical staining	Thbs4, Col 1, Gapdh, THBS4, Tenc	MenSCs manifested a strong proliferative and multipotent capacity for differentiation and differentiated into Achilles tenogenic cells	YIJING 2016	10.3892/etm.2017.4383
	Mice	α-MEM 10%FBS 1%penicillin/streptomycin	lncRNA MEG3	Whole Transcriptome Sequencing qRT-PCR Western Blot Alkaline Phosphatase Staining and Activity	AGO2 OCN, OPN, Runx2, TCF4 and β-catenin	the lncRNA MEG3 promoted osteogenic diferentiation of TDSCs and thus the formation of heterotopic ossifcation, which could be a potential therapeutic target.	Hang 2023	10.1007/s12015-023-

				Immunofuorescence<br>Immunohistochemical Staining<br>Micro-CT<br>FISH<br>RNAscope<br>RNA Immunoprecipitation (RIP)<br>RNA Pull-Down Assay				10562<br>-w
	Rat	LG-DMEM with 10% FBS	palovarotene	CCK8<br>Alizarin Red and Alkaline Phosphatase Staining<br>RT-PCR<br>Immunofluorescence<br>Micro-CT<br>Immunohistochemistry	Cell viability, OCN, SOX9, RUNX2, β-actin	palovarotene may be a novel HO inhibitor, while other drugs or antibodies targeting Smad and NF-κB signaling pathways may also prevent or treat HO.	Junchao 2022	10.1155/2022/1560943
	Human	None	Mohawk	microscopy, qRT-PCR, fow cytometry sorting, presto-blue cell viability<br>assay and immunofuorescence	CD31, α-SMA, ACTA2, Pdgfr, ANGPTL2	This study provide the first in vivo and in-vitro evidence of tendon stem progenitor cells to myofbroblasts transition and show improved tendon healing via angiofbrosis modulation, thus opening potential therapeutic avenues to treat tendinopathy patients.	Mechakra 2022	10.1038/s41598-022-24195-5
	Rat	LG-DMEM,10% FBS, and 1% penicillin–streptomycin	curcumin	CCK8, Cytokines arrays, qRT-PCR, chitosan microspheres, Radiographic evaluation, TEM, Histology, immunohistochemistry, and immunofluorescence assays	effect of curcumin, a natural anti-inflammatory agent, on regulating the	controlled-release curcumin can manipulate the fate decision of TSPCs, and that it promotes the tenogenesis and inhibits the osteogenesis of TSPCs in a pathological microenvironment, which	Chen 2019	10.1016/j.msec.2019.04.090

					differentiation of TSPCs	provides a possible new therapeutic strategy for tendon disease.		
	Rat	DMEM 10%FBS 1% penicillin–streptomycin	Inhibition of JAK-STAT Signaling Pathway	Cell Transfection，RNA Sequencing，qRT-PCR，Western Blotting，β-Galactosidase Staining，Cell Cycle Analysis，Colony Forming Unit Assays，EdU Detection，CCK-8，Population Doubling Time Assay，TSPCs Migration Assay，Investigation of Actin Dynamics	JAK2 ，p-JAK2, STAT3, p-STAT3, p16INK4A ，cyclin D1, cyclin B1, and GAPDH	Pharmacological inhibition of JAK STAT signaling pathway with AG490 similarly attenuated cellular senescence and senescence-associated secretory phenotype (SASP) of aged TSPCs. In addition, inhibition of JAK-STAT signaling pathway also restored the age-related dysfunctions of TSPCs, including self-renewal, migration, actin dynamics, and stemness.	Chen 2021	10.3389/fcell.2021.650250
	Rat	LG-DMEM 10% FBS, 100 U/ml penicillin, 100 mg/ml streptomycin, and 2 mM l-glutamine	heterotopic ossification	qRT-PCR，Immunohistochemistry Staining，Western Blot，Osteogenic Differentiation Assay	BMP-2/4/7，Osteogenesis-Related Genes Expression，Noggin，Runx2, OPN, and OCN	enhanced osteogenic differentiation of TSPCs contributes to the increased heterotopic ossification in aged tendon, which might be induced by the higher expression of BMPs with aging.	Dai 2020	10.3389/fcell.2020.570605
	Rat	DMEM with 15%FBS 1% penicil lin/streptomycin	VEGF	PCR，western blot，histological score and bio mechanical property，histo chemical	IL-6, IL-10, and TNFα IL-10, G-CSF, and VEGF	VEGF not only plays an important role in decreasing adi pocyte accumulation but also improves vascularization of the tendon during aged tendon healing. We believe active regulation of VEGF may improve the treatment	Lai 2022	10.1096/fj.202200213r

						of age-related tendon diseases and tendon injuries.		
	Human	None	TGFb	TEM, hybridization and immunohistochemistry, staining	ScxGFP, TGFb	recruited cells originated from a Sox9-expressing lineage and their recruitment was dependent on cell autonomous TGFb signaling. The cells identified in this study thus differ from previous reports of cell recruitment into injured tendons and suggest a critical role for TGFb signaling in cell recruitment, providing insights that may support improvements in tendon repair.	Guak-Kim 2021	10.1016/j.stemcr.2021.10.018
	Rat	DMEM 20%FBS 100 U/ml penicillin and 100 μg/ml streptomycin	rapamycin	Western Blot and qRT-PCR. microplate reader, Semi-Quantification of Histochemical and Immunohistochemical Staining	LPL, PPARγ, SOX-9, collagen II, Runx-2, and osteocalcin genes,p-S6,S6,p-4EBP1,4EBP1,β-actin	these findings suggest that mechanical loading activates the mTOR signaling in TSCs, and rapamycin may be used to prevent tendinopathy development by blocking non-tenocyte differentiation due to mechanical over-activation of mTOR in TSCs.	Nie 2021	10.3389/fcell.2021.687856
	Human	DMEM 10%FBS 1% penicillin and streptomycin	p16/miR-217/EGR1	RT-PCR<br>Western blot<br>Enzyme-linked immunosorbent assay<br>Senescence-associated β-galactosidase staining	Scx, Tnmd, Bgn, Dcn, Col1, and Col3.	These results indicated that p16 inhibits tenogenic differentiation of TSPCs via microRNA signaling pathways, which may serve as a potential target for the prevention or treatment in the future.	Han 2017	10.1093/abbs/gmx104

				Lentivirus transduction and miRNA transfection<br>Plasmid construction and dual-luciferase reporter<br>assay				
	Rat	DMEM 15%FBS<br>1% penicillin and streptomycin	PPARγ	qRT-PCR, western-blot and immunofuorescent staining	PPARγ,CCAAT,C/EBPα, FABP4, p16ink4a, Perilipin, Angptl-4, Slc27a6	aging inhibited adipogenesis of TSPCs by down-regulating PPARγ signaling. It is not<br>likely that the adipocyte accumulation in aging tendon during repair was due to the aging of TSPCs. This may provide<br>new targets for curing aging tendon injuries or tendinopathies.	Lai 2021	10.1186/s13018-021-02720-y
	Mice	DMEM 10%FBS<br>1% penicillin and streptomycin	Tenomodulin	Genotyping，RNA isolation and PCR，WB，ELISA	Tnmd，b-actin	loss of Tnmd in mTSPCs led to profoundly altered gene expression profile, insufficient adhesion to collagen type I, and impaired ability to contract the extracellular matrix.	Yin 2019	10.1016/j.bbrc.2019.03.063
	Rat	DMEM 10%FBS<br>1% penicillin and streptomycin	treadmill exercise	histology and immunohistochemistry	distal tendon (DT) mid/proximal tendon (MPT)	Daily moderate exercise (treadmill running) mainly improves in vivo cell proliferation in rapidly proliferating cells, whereas the stem/progenitor pool remains constant.	Eva 2013	10.1007/s00167-013-2446-7

	Rat	None	Nucleostemin- and Oct 3/4-positive stem/ progenitor cells	Histology and immunohistochemistry，Image calculation	Oct 3/4，nucleostemin，Dyn 2，CD45	Oct 3/4 may thus act as a more local, migrating stem/progenitor cell involved in injury-site-specific regenerative effects, as compared to the more general proliferative role of nucleostemin-positive stem/progenitor cells.	Eva 2015	10.1186/s12891-015-0658-3
	Rabbit	DMEM + 10 % FBS	leukocyte-containing and pure platelet-rich plasma (PRP)	Immunostaining proliferation assay，Cell morphology，qRT-PCR，Western blot	collagen, MMP-1, and MMP-13，IL-1β, IL-6, and TNF-α，PGE2	These findings indicate that, while both L-PRP and P-PRP appear to be "safe" in inducing TSC differentiation into active tenocytes, L-PRP may be detrimental to the healing of injured tendons because it induces catabolic and inflammatory effects on tendon cells and may prolong the effects in healing tendons.	Zhou 2015	10.1186/s13287-015-0172-4
	Rat	Not given	N-acetyl-L-cysteine	cell counting kit-8, fuorescence staining, Western blotting, and immunofuorescence,	COL1A1, TNC, SCX, and TNMD	NAC treatment promoted the survival and diferentiation of TSPCs to facilitate tendon repair after ten don injury in rats. Thus, NAC may be valuable for the treatment of tendon injury	Lu 2023	10.1186/s12860-022-00463-0
	Human	DMEM,1% MEM-Amino-acids 10% FBS, and 1% L-ascorbic acid-2-phosphate	Activation of EphA4 and EphB2	qPCR,Cytochemistry,Self-Renewal Analysis,Western Blot,Migration Analysis,Quantification of Cell Area and Actin Dynamics	EphA4,EphB2,EphB4,EFNB1 and EFNB2,phospho-FAK,FAK,total and phospho-ERK1/2,total and	decreased expression of ephrin receptors during tendon aging and degeneration limits the establishment of appropriate cell-cell interactions between TSPC and significantly diminished their proliferation, motility, and actin turnover.	Cvetan 2015	10.3389/fnagi.2015.00246

					phospho-Akt,total and phospho-p38,total and phospho-Jnk,GAPDH	Taken together, we could propose that this mechanism might be contributing to the inferior and delayed tendon healing common for aged individuals.		
	Human	α-MEM<br>10 % FBS<br>1 % A/A solution	tenomodulin	immunomagnetic bead separation<br>RT-PCR	EGF, bFGF, TGF-β1 and PDGF-BB，TNMD，scleraxis, tenomodulin, tenascin C and decorin，Collagen I and Collagen III	The results obtained indicated that TNMD+ cells exhibit phenotypical features of tendon progenitor cells and can be biochemically induced towards tenogenic lineage, demonstrating that this subset of hASCs can provide a reliable source of progenitor cells for therapies targeting tendon regeneration.	Gonçalves	10.1002/term.2495
	Human	α-MEM<br>10 % FBS<br>1 % A/A solution	Ectopic Expression of Scleraxis	Analysis of cell area<br>Self-renewal analysis<br>RT-PCR<br>Western blot<br>ELISA<br>Hydroxyproline assay<br>Luciferase assay<br>Cell differentiation<br>Immunocytochemistry	FLAG M2<br>Scx<br>β actin	These resulted showed a remarkable upregulation of the T/L differentia tion gene Tnmd in hMSC-Scx. From these results, we conclude that Scx delivery results in the direct program ming of hMSC into tendon progenitors and that the newly generated hMSC-Scx cell line can be a powerful and useful tool in T/L research.	Paolo 2012	10.1089/scd.2011.0150
	Rat	DMEM with 10%FBS	degenerative tendon microenvironment	Immunofluorescence staining, H&E staining, real-time PCR, and Western blot	COL1 and TNMD<br>COL2, SOX9, Runx2, and ALP, FAK and ERK1/2	this study found that the degenerative tendon microenvironment induced TDSCs to differentiate into	Liu 2018	10.1155/2018/2613821

						chondrogenic and osteogenic lineages. It could be attributed to the cell morphology changes and reduced FAK and ERK1/2 activation in the degenerative microenvironment of tendinopathy		
	Rat	LG-DMEM<br>10%FBS<br>100 U/ml penicillin, 100 mg/ml streptomycin, and 2 mM L-glutamine	TDSCs	Animal Surgery<br>Histology<br>Biomechanical Test<br>Ultrasound Imaging<br>Ex Vivo Fluorescence Imaging	CD90 and CD73 Oct-4 SSEA-4, and nucleostemin,	In conclusion, TDSCs promoted earlier and better repair in a rat patellar tendon window defect model.	Ni 2012	10.1002/jor.21559
	Rat	DMEM<br>10% FBS<br>antibiotics	TDSCs	qRT-PCR<br>Sirius red staining	CD73， CD90， EphA4， Col3a1， Lox， TenC， Col1a1， Tnmd， Dcn， Fmod	This study identified that TDSCs had the potential of spontaneous tenogenic differentiation, which may be a better cell source for the treatment of tendon injury.	Guo 2015	10.1016/j.yexcr.2016.01.007
	Human	Not given	Adipose-derived stem cell exosomes	Histological examination<br>RT-PCR<br>Biomechanical testing	RUNX2, Sox-9, TNMD, TNC and Scx and the mechanical properties of the articular portion	The ADSC-exos have the potential to promote the rotator cuff repair by mediating the TDSCs.	Fu 2021	10.2217/rme-2021-0004
	Human	α-MEM with 2 mM glutamine,1% antibiotic-	hypoxia inducible factor (HIF)	Western Blot, trypan blue dye exclusion assay, MTT Assay, real-time PCR	HIF-1α<br>Lamin A/C	These results support the notion that hypoxia, by activating HIF, plays a crucial role in preserving stem cells in an	Alessandra 2018	10.1155/201

		antimycotic mixture,20% FBS				undifferentiated state in the "hypoxic niches" present in the tissue in which they reside before migrating in more oxygenated areas to heal a damaged tissue.		8/9468085
	Human	DMEM with 10% FBS and 1% penicillin–streptomycin, and 2 mM L-glutamine	Pin1 silence	β-gal, Telomerase activity, RT-PCR, WB	Pin, P16, CD44, CD90, Stro-1, CD34	Pin1 siRNA transfection promoted senescence in TSPCs.	Chen 2015	10.1016/j.bbrc.2015.06.163
	Rat	DMEM with 10% FBS and 1% penicillin–streptomycin, and 2 mM L-glutamine	miR-135a	RT-PCR, β-gal, WB,<br><br>Luciferase assay	ROCK1, P16, GAPDH, miR135a	This study suggest that miR-135a plays an important role in TSPC senescence via targeting ROCK1.	Chen 2015	10.1016/j.bone.2014.11.001
	Rat	LG-DMEM with 10% FBS and 1% penicillin–streptomycin, and 2 mM L-glutamine	AMPK inhibition/mTOR activation	Proteomics Analysis, Cell Migration Assay, Cell Proliferation Assay, β-Gal Staining<br><br>Western Blot Assay, RT-PCR and animal experiment	pAMPK, AMPK, pmTOR, mTOR, p-S6K1, S6K1, beta-actin, P16, P53	This study revealed new insight and mechanistic exploration of TSPC senescence and proposed a novel therapeutic treatment for age-related tendon disor- ders by targeting the AMPK/mTOR axis at the early stage of aging.	Dai 2023	10.1007/s12015-023-10526-0

	Rat	DMEM containing 10% FBS, 100 U/mL penicillin, 100 mg/mL streptomycin and 2 mM L-glutamine	FOXP1 inhibition	WB, β-Gal Staining, CCK-8, RT-PCR, migration assay, Colony forming unit (CFU) assays	P16, cell viability, FOXP1, GAPDH,	These results indicate that FOXP1 plays a crucial role in TSPCs aging	Xu 2018	10.1016/j.bbrc.2018.08.136
	Rabbit	LG-DMEM with 10% FBS, and antibiotics (penicillin 100 U/mL, streptomycin 100 g/mL)	SOX11 over expressing	CCK8 Assay, Alizarin red S staining, qRT-PCR, In Vitro Tube Formation Assay, Western Blot, immunofluorescence (IF), animal experiment	osteogenic differentiation-related genes: OCN ,Runx2, angiogenesis-related genes :VEGF, BMD Index	TDSCs over-expressing Sox11 might be a promising cell source for stem cell therapy to promote bone regeneration, such as ONFH, fracture, bone defect, and so on.	Ni 2021	10.1177/0963689721105387 0
	Human	DMEM with 10%FBS, and 1% penicillin/streptomycin	Celecoxib	CCK8 Assay, Cell Viability Assay(live/dead cell staining kit), SA-β-gal Staining, qRT-PCR，Immunofluorescence(IF), Western Blot, sirius red staining, Alcian Blue Staining	osteogenesis, adipogenesis, and chondrogenesis analysis: Alizarin red S staining, alcian blue staining, and oil red O staining	Celecoxib treatment can prevent inflamm-aging–induced TDSC senescence, which holds potential for alleviating the development of degenerative RCT.	Cai 2022	10.1177/03635465221098133

	Human	Osteogenic-induced medium (containing 50 µM ascorbic acid, 10 mM β-glycerophosphate, and 100 nM dexamethasone)	LncRNA AC108925	Alkaline phosphatase (ALP) staining, Alizarin red S (ARS) staining, qRT-PCR, Western blot, shRNA transfection	osteogenic markers: Runx2, ALP and OCN	Targeting the AC108925/miR146a-3p axis might be a latent way to treat tendinopathy.	Liu 2022	10.1016/j.prp.2022.154230
	Human	a-MEM, 2 mM glutamine, 100 IU/mL penicillin, 100 mg/mL streptomycin, and 20% FBS.<br><br>Hypoxic conditions (0.5%, 5%, 10% O2)	Hypoxic	CCK8 Assay, qRT-PCR, Multi-differentiation assays(Oil Red O, Alizarin Red, Alcian Blue), Western blot	levels of stem cell markers: NS, Nanog, Oct-4, and SSEA-4, PPAR-γ(adipogenic marker), Runx-2 (osteogenic marker), Sox-9 (chondrogenic marker)	Hypoxic condition encouraged self-renewal capacity of hTSCs, but inhibited their multi-differentiation potential. Moreover, excessively low oxygen concentration impaired the capacity of hTSCs.	Yu 2017	10.12659/msm.903892
	Human	DMEM/Ham's F-12 (1:1mixture),glutamine,1 × MEM amino acids,10% FBS and 1% L-ascorbic acid-2-phosphate	CITED2 Downregulation	Multipotent differentiation assays, Population-doubling and colony-forming unit assays(0.5% crystal violet/methanol staining), qRT-PCR, Western blot, WST-1 analysis, Senescence analysis (β-gal), Immunofluorescence(IF), CITED2 small hairpin RNA	the tendon-related genes: SCXand TNMD,<br>the cell cycle gene regulators: MYC and p21	The downregulation of CITED2 contributes to TGFβ-mediated senescence providing an insight into the molecular and cellular mechanisms that contribute to tendon aging and degeneration, which may aid the development of cell-based therapies for tendon repair	Hu 2016	10.1007/s00441-016-2552-1

	Rat	DMEM, 10% FBS, 1% penicillin/streptomycin	PRP, TDSC	Flow cytometry assay, Western blots, RT-PCR, Animal Experiments, Biomechanical testing	Histology scores, the biomechanical properties of Achilles tendon tissue, tenocyte-related genes:Col1, SCX and Tenascin C, Non-tenocytegenes:Runx2,PPARγ, SOX9	The PRP induced, FAK and ERK1/2 dependent activation of tenocyte related genes in TDSCs in vitro of the PRP with TDSC combination might occur by means of an improved TDSC differentiation toward the tenocyte lineage.PRP combined with TDSCs is potentially effective for the treatment of tendinopathy.	Chen 2014	10.1159/00036965 9
	Rat	DMEM, 20% FBS, 1%penicillin/streptomycin	Glucose, MIF	RT-PCR, Small Interference RNA Transfection for TdSCs, Animal Experiments	Tenogenic differentiation markers: Egr1, Scx, type 1 collagen, and Tnmd, Osteochondrogenic differentiation markers (aggrecan, bone morphogenic protein (BMP)-2, and Sox9,,Pain-related substances: CGRP, TRPV	Tendon homeostasis could be affected by hyperglycemic conditions, and MIF appears to alter the differentiation of TdSCs via enhancement of the osteochondrogenic differentiation in hyperglycemic conditions.	Kim 2021	10.3390/ijms22168983

	Mice	alpha-MEM, 2mM L-glutamine, antibiotics/antimycotics, 100mM 2-mercaptoethanol, and 20% fetal bovine serum	Regional Differences	Animal Experiments, Colony-forming unit assay, Flow cytometry, qRT-PCR, Histological staining, Immunohistochemistry	tendon markers：Tnmd, Scx, vascular marker: Emcn, pericyte marker: Cd133	Intrinsic repair may require a progenitor class with predominant tendon marker expression, while extrinsic repair may involve a progenitor class recruited from perivascular cells of the peritenon.	Mienaltowski 2012	10.1089/ten.tea.2012.0182
	Human	DMEM, 1% penicillin-streptavidin-glutamine (PSG),10% FBS	celastrol	Proliferation assay, Multi-differentiation assays, qRT-PCR, Western blot, Luciferase assay, Chromatin immunoprecipitation (ChIP)	adipogenic marker: PPARγ, chondrogenic marker: Sox9, osteogenic marker: Runx2, Colony number, colony size, and proliferation of hTSCs	The positive effect of celastrol on the stemness of hTSCs and elucidated the essential role of the HIF1α-Smad7 pathway in this process.	Wu 2017	10.1186/s13287-017-0724-x
	Rat	alpha-MEM, 2 mM L-glutamine, antibiotics/antimycotics, 100 mM 2-mercaptoethanol, 20% fetal bovine serum	BMP12，CTGF	Western blot，Cell immunofluorescence assays，qRT-PCR，CCK, Animal experiments，Histological assessments，Biomechanical testing	tenogenic differentiation genes：type I/III collagen, tenascin-C, and scleraxis，osteogenic, adipogenic, chondrogenic	BMP12 and CTGF transfection stimulate tenogenic differentiation of TDSCs. The synergistic effects of simultaneous transfection of both may significantly promoted rat patellar tendon window defect regeneration.	Xu 2017	10.1016/j.jbiomech.2017.11.004

					markers：Runx2, PPAR, SOX-9			
	Human	low-glucose DMEM, 10% FBS, 100 U/ml peni cillin, and 100 mg/ml streptomycin	LncRNA MALAT1	Animal experiments, HE staining, Immunohistochemistry, Cell transfection, Western blot, RT-qPCR, Sirius Red staining, Bioinformatics analysis, Dual-luciferase reporter assay	Tendon related transcription factors and marker genes :SCX, Mkx, COL1a1, Fmod, Mmp3 and Thbs4	Targeting the MALAT1/miR-378a-3p/MAPK1 axis may be a promising avenue for the treat ment of tendinopathy.	Zhao 2022	10.1080/21655979.2022.2076507
	Rat	Dul- becco's modified Eagle's medium, 10% fetal bovine serum, 100 U=mL penicillin, 100 mg=mL streptomycin, 2 mM L- glutamine	BMP-2	qRT-PCR, Alizarin red S staining o, H&E staining, alcian blue staining	Tenogenic Marker: Col1a1, Scx, Tnmd, Osteogenic,Adipogenic,Chondrogenic Differentiation Marker: Alpl, Runx2, PPARγ, C/EBPα	BMP-2 promoted GAG deposition, aggrecan expression, and enhanced non-tenocyte differentiation of TDSCs in vitro. The effect of BMP-2 on TDSCs might provide insights into the histopathological changes of tendinopathy	Rui 2012	10.1002/jor.22290
	Rat	DMEM/F12, 10% FBS, 100U/ml penicillin, and 100 μg/ml streptomycin	Proanthocyanidins	Flow cytometry assay,Osteogenic Differentiation Assays,Adipogenic Differentiation Assays,Chondrogenic DifferentiationAssays,CCK-8,Antioxidant Test, Real-Time PCR, Western Blot	Oxidative damage markers: Nrf-2,GCLM, HO-1, NQO-1	PCs could protect against the oxidative damage induced by H2O2 in TDSCs, and the cytoprotective effects might be due to the ability of PCs to activate the expressions ofGCLM, HO-1, and NQO-1 via upregulating Nrf-2 signaling pathway	Sun 2017	10.1155/2017/7529104
	Human	DMEM High Glucose, 10% fetal bovine serum, 5%	Curcumin, Hyaluronic Acid, Palmitoylethanola	Trypan Blu staining, Immunocytochemistry, von Kossa and Alcian blue staining, CCK8	cell survival	The influence of several drugs or supplements used for the treatment of musculoskeletal disorders should be taken	Meglio 2020	10.32098/mltj.03.

		horse serum, 0.2 mM glutathione, 10 ng/ml β-FGF, erythropoietin 5 UI, porcine gelatine 50 g/ml, penicillin 10,000 U and streptomycin 10 mg/ml	mide, Diclofenac sodium, Triamcinolone acetonide and Thiocolchicoside			into consideration in order to take the full advantage of the healing properties of stem cells within tendons.		2020.04
	Rat	Low-glucose DMEM, 10% fetal bovine serum, 100 U/ml penicillin and 100 mg/ml streptomycin	Magnesium	Alizarin red staining, Real-time PCR, Adenosine 50-triphosphate assay, Mitochondrial isolation and calcium fluorescence imaging, Mitochondrial membrane permeability assessment, JC-1 assay and flow cytometry	Matrix mineralization, the fluorescence intensity of the mitochondria	The balance between Mg2þ and Ca2þ influences mitochondrial calcium exportation and provides another explanation for the mechanism underlying matrix calcification in TDSCs.	Yue 2016	10.1016/j.bbrc.2016.06.108
	Rat	DMEM/F12, 20% FBS	hypoxic	CCK8, Alizarin red staining assay, Alkaline phosphatase (AKP) activity detection, real-time PCR, Western blotting	Cell viability, cell proliferation, AKP activity, Gene expression:ALP, osteocalcin, collagen I, RUNX2	Normoxic culture promotes osteogenic differentiation of rTDSCs compared with the hypoxic culture, and the ERK1/2 signaling pathway is involved in this process	Li 2016	10.7150/ijms.16045

	Human	DMEM, 10% FBS, 1% penicillin/streptomycin	long non-coding RNA LINCMD1	Western blot, Cell osteogenic differentiation assay, Lentivirus transduction, Qrt-PCR, XTT cell proliferation assay,Dual-luciferase reporter assay, RNA immunoprecipitation assay	tendon-related genes: SCX, TNMD, DCN, and Collagen I, osteogenic marker: RUNX2, ALP	The induction of LINCMD1 in tenogenic differentiation of hTDSCs through miR-342-3p/EGR1 axis.	Qu 2023	10.1080/03008207.2023.2217258
	Rat	DMEM, 10% FBS, 100 units/mL penicillin, 100μg/mL streptomycin	Spironolactone	Identification ofTrilineage Differentiation Potential,Flow Cytometry, CCK-8, Immunofluorescence, qRT-PCR, β-Galactosidase Activity Assay, ROS Detection, Western Blot, Animal experiments, Histological Analysis, Immunohistochemistry Analysis, X-Ray	Inflammation-related mediators: iNOS, COX2, MMP13, and MMP9, senescence crucial protein: p53, Autophagy biomarkers: ATG5, ATG7, and Beclin-1	The protective role of SP on the pathological process of tendinopathy both in vitro and in vivo, indicating a potential therapeutic strategy for tendinopathy treatment.	Xu 2021	10.1155/2021/5519587
	Rat	LG-DMEM, 10 % fetal calf serum (FBS), 50 μg/ml penicillin, 50 μg/ml streptomycin, and 100 μg/ml neomycin	TNF-α, TGF-β1	Flow cytometry, Multi-lineage differentiation potential, CCK-8, Real-time RT-PCR, Western blotting	tenogenic/osteogenic-related marker: Scx, Tnmd, Col1A1,Alpl and Runx2, proliferation of TDSC	Combining the use of TNF-a and TGFb1 could improve the proliferation and differentiation of TDSC in vitro, and the expression of I-Smad is negatively correlated with TDSC proliferation and differentiation.	Han 2016	10.1007/s10529-017-2296-3
	Mice	low-glucose DMEM ,10% FBS,	fibrosis transmembrane	two-dimensional electrophoresis, mass spectrometry, Western	annexin A1, inflammation	Decreased annexin A1 expression could contribute to the elevated inflammation in	Liu 2018	10.1002/prc

		100U/ml penicillin,100mg/ml streptomycin	conductance regulator (CFTR)	blotting, qRT-PCR, Immunofluorescence and immunohistochemistry staining, Animal experiments	markers: COX-2, IL-6	DF508 mice during tendon injury, wich could be considered as a new potential biomarker or drug target for a possible therapeutic approach in clinical practice.		a.2017 00162
	Human	L-DMEM, 1% penicillin-streptomycin, 10% fetal bovine serum	reactive oxygen	nanoparticle morphology, Beckman DelsaTM Nano analyzer, CCK8, alizarin red staining, alkaline phosphatase staining, Western blotting, real-time PCR, ROS measurement, HE staining, Immunohistochemistry and immunofluorescence	ACAN , OPN , OCN , MMP13 , β-actin, Col1, OPN, MMP13, IL-1β	This work demonstrated that sustained release of GA targeting ROS and ectopic ossification is a practical therapeutic strategy for treating tendinopathy.	Shen 2022	10.10 16/j.ac tbio.2 022.0 9.007
	Mice	α-MEM containing 20% FBS, 2 mM L-glutamine, 100 μM 2-mercaptoethanol and 1% penicillin–streptomycin	Wnt5a knockdown	RNA sequencing, WB, β-gal staining, RT-PCR, Cell cycle analysis,	Wnt5a, GAPDH, beta-catenin, Axin2, Lgr5, p16, Tubulin, p-Jak2, Jak2, p-STAT3, STAT3,	These results showed a critical role of noncanonical Wnt5a signaling in TSPCs senescence, and Wnt5a could be an attractive therapeutic target for antagonizing tendon aging.	Chen 2021	10.118 6/s132 87-021-02605 -1
	Rat	LG-DMEM containing 10% FBS and 1% antibiotic	CTGF	IF, CFU-F assay, Multilineage differentiation, In vitro cell proliferation, Animal experiments, Histology and histomorphometry	CD146, CD31, Histology, Stiffness, Col1a1, Col3a1, Tnc, Vim, Tnmd, Scx, Ki67, p-FAK, p-ERK1/2	This study support the use of endogenous stem/progenitor cells as a strategy for tendon regeneration without cell transplantation and suggest this approach warrants exploration in other tissues.	Lee 2015	10.117 2/JCI8 1589

	Human	DMEM with 10% FBS	Extracorporeal Shock Wave Treatment	IF, Flow cytometry analysis, Multidifferentiation, RT-PCR	CD146, CD44, CD90.2, CD105, CD34,	ESWT significantly accelerated hTSPCs differentiation, suggesting that the clinical benefits of ESWT may be ascribed to increased efficiency of tendon repair after injury.	Leone 2016	10.18632/oncotarget.7064
	Rat	DMEM supplemented with 20% FBS 100 U/ml penicillin, and 100 μg/ml streptomycin	Bleomycin and Rapamycin	β-gal, WB, RT-PCR, IF, CCK-8	Cell Proliferation, p62, LC3I, LC3II, p-S6, S6, p53, p21, beta-actin,	By reducing TSC senescence, rapamycin may be used as a therapeutic to inhibit tendinopathy development in the aging population by promoting autophagy.	Nie 2021	10.1155/2021/6638249
	Rat	DMEM containing 10% FBS, 1% penicillin-streptomycin	AQP1 knock down	Microarray analysis, IF, wounder healing, WB, β-gal, CCK-8, RT-PCR, Cell cycle analysis, Colony-forming unit (CFU) assays,	Cell Proliferation, AQP1, GAPDH, P16, Cyclin D1, Cyclin B, Cyclin A, Scx, Tnmd, Bgn, Mkx, ColA1, Nestin	This study demonstrated the critical role of AQP1 in the regulation of TSPCs senescence and provided a novel target for antagonizing tendon aging.	Chen 2020	10.1038/s41419-020-2386-3
	Rat	LG-DMEM with 10% FBS and 50 mg/mL penicillin, 50 mg/mL streptomycin,100 mg/mL neomycin	TGIF1 siRNA	Chondrogenic differentiation assay, IHC, WB, RT-PCR, Immunoprecipitaes, animal experiment	Chondrogenic differentiation, TGIF1, beta-actin, Sox9, Aggrecan, ColII, Scx, Tnmd, ColI, Smad2, p-Smad2	the tendon-derived stem cell modified with TGIF1 gene silencing has promising effects on tendon-to-bone healing which can be further explored as a therapeutic tool in regenerative medicine.	Chen 2015	10.1159/000438568

	Rat	LG-DMEM with 10% FBS 50 µg/mL penicillin, 50 µg/mL streptomycin, and 100 µg/mL neomycin	TDSC-Exos	miRNA library construction and sequencing, Bioinformatic analysis, Dual-luciferase reporter assay,	CD106, CD31, CD90, CD44,CD9, CD63, CD81, TSG101	TDSC-Exos enhanced tenon repair through miR-144-3p-regulated tenocyte proliferation and migration. These results suggest that TDSC-Exos can serve as a promising strategy to treat tendon injuries.	Song 2022	10.1186/s13287-022-02723-4
	Rat	LG-DMEM with 10% FBS	Transduction of Scx into TDSCs	RT-PCR, Ex vivo Fluorescence Imaging, vivaCT Imaging, Histology, Biomechanical Test, IHC	Scx, Col1A1, Eya1, Acan, Col2a1, Runx2, Thbs4, Tnc, Col1a1, Epha4, Six1, Eln, Tnmd, Bgn, Sox9, Biomechanical result, histology result	The transplantation of GFP-TDSC-Scx promoted healing at the early stage of tendon repair in a rat patellar tendon window injury model.	Tan 2014	10.1371/journal.pone.0097453
	Human	LG-DMEM with 10% FBS and 50 µg/mL penicillin, 50 µg/mL streptomycin, and 100 µg/mL neomycin	Hypoxia	Colony-forming unit assay, 5-bromo-2′-deoxyruidine assay, Alamar blue assay, β-gal assay, Immunophenotypes, Multilineage differentiation potential, Expression of tendon-related markers	Cell viability, CD44, CD73, CD 90, and CD105 ,CD34, CD45, CD146, and Stro-1 and expression of tendon-related markers	hypoxia is advantageous for efficient expansion of hTDSCs in vitro for tendon tissue engineering.	Lee 2011	10.1089/ten.TEA.2011.0130

	Rat	DMEM with 10% FBS and 1% penicillin-streptomycin	Leucine rich repeat containing 32	Animal experiment, Multilineage differentiation potential , Histologic, Histochemical, IF, WB,	CD29, CD44, CD45, CD90, lrrc32, beta-actin, Tnmd, TGF-beta1, Smad2/3, p-Smad2/3	These findings indicated that Lrrc32 promoted the tenogenic differentiation of TDSCs in vivo.	Kang 2023	10.1538/expanim.22-0009
	Rat	LG-DMEM with 10% FBS and 100 U mL−1 penicillin and 100 μg mL−1 streptomycin	dimethyl oxalylglycine (DMOG)	Animal experiment, RT-PCR, WB, Histological Evaluation, Biomechanical Testing	HIF-1a, EGR1, beta-actin, ColII, Sox9, Aggrecan, ColI, Ten-C, SCX, Biomechanical result, Histological result	DMOG improves TDSC differentiation ability, and transplanting TDSCs pre-treated with DMOG into rats with Achilles tendon injury can effectively improve tendon regeneration, which may serve as a novel approach for the treatment of Achilles tendon injury.	Wang 2023	10.1002/adtp.202200164
Tissue Engineering	Rat	Not given	None	a 36o YX-LiTaO3-based Love wave sensor with a parylene-C wave guiding layer was adopted as a cell-based biosensor to monitor the adhesion process of TDSC	Sensor data	the Love wave biosensor as a very promising sensor platform for investigating cellular activities under multiple physiological conditions.	Wu 2018	10.1007/s00249-019-01349-4
	Rat	DMEM with 10% FBS and 1% penicillin/streptomycin	PLGA/IO MPs-PLL	MR，Photoacoustic，Iron quantification，histological analysis	TSC proliferation and ability，concentration of PLGA/IO, load to failure	PLGA/IO particle was a promising dual-modal MR/PA contrast for noninvasive long-term stem cell tracking.	Cheng 2020	10.1038/s41598-020-69214-5

	Rat	DMED with 10% FBS,100 U/ml penicillin, 100 mg/ml L-glutamine	Co-culture BMSCs and TDSCs	Immunofluorescent staining, CCK-8, wounder healing, WB, RT-PCR	SCX, PPAR, GAPDH, RUNX2, SOX9, CD44, CD90, cell viability and migration ability et al.	Tenascin C has a significant impact on the proliferation and differentiation of co-cultured BMSCs and TSCs. IGF-IR, ROCK, and MEK may become involved in the process after TNC treatment.	Liu 2019	10.1515/biol-2019-0063
	Rat	LG-DMEM with 10% FBS and 50 μg/ml penicillin, 50 μg/ml streptomycin 100 μg/ml neomycin and connective tissue growth factor (25 ng/mL) and ascorbic acid (25 mM)	Cell Sheet for ACL reconstruction	CT, Biomechanical Testing<br><br>Histology and Immunohistochemistry Staining	ultimate load and stiffness and GFP	The TDSC sheet improved early graft healing after ACL reconstruction in the rat model.	Lui 2014	10.1177/0363546513517539
	Rat	LG-DMEM with 10% FBS and 50 μg/ml penicillin, 50 μg/ml streptomycin 100 μg/ml neomycin	TDSC transplant to tendon injury situ	IHC, Histology, Ex vivo fluorescence imaging	GFP, CD68, CD163, mast cell tryptase	allogeneic TDSCs promoted tendon repair in the medium to long term and exhibited weak immunoreactions and anti-inflammatory effects in the hosts after transplantation in a rat model	Lui 2014	10.1089/ten.tea.2013.0713
	Rat	LG-DMEM with 10% FBS and 50	CTGF and ascorbic acid	Histology Ultrasound imaging ,vivaCT	Histological scoring	Transplantation of TDSCs promoted tendon repair up to week 16, with pre-	Lui 2016	10.1016/j.jc

		μg/ml penicillin, 50 μg/ml streptomycin 100 μg/ml neomycin and CTGF (25 ng/mL) and ascorbic acid (25 μmol/L)	treated TDSC transplantation	imaging ,Biomechanical test Immunohistochemistry ,Scanning electron microscopy	Ectopic mineralization，Biomechanical properties， GFP and PCNA	treatment of TDSCs with CTGF and ascorbic acid showing the best results up to week 8 after injury in a rat patellar tendon window wound injury model.		yt.2015.10.005
	Human	DMEM/F-12 with 10% FBS and 1% ascorbic acid I and 1% MEM amino acids I and 1% dual antibodies	3D RADA nanofiber hydrogel	Animal experiment, hind limb function test, Histological and immunohistochemical staining,	Function score, cell density and nuclear orientation	the combination of TSPC and nanofiber hydrogel provide an optimistic alternative method to accelerate functional tendon repair with reduced heterotopic ossification.	Zhang 2023	10.1089/ten.TEA.2022.0183
	Human	DMEM with 10% FBS and 1% penicillin–streptomycin	decellularized matrices	Fluorescence-activated cell sorting<br><br>Multipotent differentiation<br><br>SEM, CCK-8, ALP assay, IF, RT-PCR, animal experiment, Histological evaluation and Mechanical testing	Proliferation ability, Multipotency, histological assay results	the potential of decellularized matrix for future tissue engineering applications, as well as developing a practical strategy for functional tendon regeneration by utilizing TSPCs combined with tendon-derived decellularized matrix.	Yin 2013	10.1016/j.actbio.2013.07.022
	Rat	α-MEM with 10% FBS	YAP loaded exosome	Multipotent differentiation<br><br>Colony formation unit fibroblast	YAP1, OCT4, SOX2, SCXA, TNMD, TNC, GAPDH, P53, P21, Col1a1,	PLT-Exo-Yap1-functionalized GelMA promotes the rejuvenation of TSPCs to facilitate functional tendon regeneration	Lu 2023	10.1016/j.actbio.2023.02.018

				IF, RT-qPCR<br><br>WB				
	Rabbit	LG-DMEM with 10% FBS and 1% penicillin-streptomycin	silk–collagen sponge scaffolds	Colony Forming Unit, Multipotent Differentiation Potential, Flow Cytometry Analysis, Animal experiment,Histological Assessment	Colony formation, Multipotency, histological assay Collagen I,Collagen III, Biglycan, Tenascin Decorin, GAPDH	allogenous TSPC-seeded knitted silk–collagen sponge scaffolds can be a clinically useful application for tendon tissue engineering.	Shen 2012	10.3727/096368911X627453
	mice	LG-DMEM with 20% FBS	small molecules and 3D printing hydrogel	image-based, high-throughput screening,High-throughput qRT-PCR, IF, animal experiment, Histological, and IHC,	proliferation, tenogenesis initiation and maturation phases, tendon related gene: Scx, Tnmd, Thbs4, Col1a1, Col3a1, histological assay	The stepwise culture system for TSPCs and construction of engineered tendon grafts can not only serve as a platform for further studies of underlying molecular mechanisms of tenogenic differentiation, but also provide a new strategy for tissue engineering and development of novel therapeutics for clinical applications.	Zhang 2021	10.1016/j.biomaterials.2021.120722
	Mice	DMEM containing L-glutamine, 1 % penicillin/streptomycin/fungizone, and 10 % fetal bovine serum.	Modular, fiber-reinforced DexVS hydrogels.	Phalloidin, Hoechst 33 342, Ki67, COMSOL Multiphysics, Computational model predicts release kinetics from hydrogel-encapsulated microgels.	TGF-β1, SDF-1α, PDGF-BB, Sca-1, ScxGFP, the volume of cell outgrowth, cell migration, Gradient of chemokine concentration	Fiber reinforced hydrogels can drive the recruitment of endogenous progenitor cells relevant to the regeneration of tendon and, likely, a broad range of connective tissues.	Kent III 2022	10.1002/adfm.202220755

	Mice	alpha-MEM, 20% fetal bovine serum, 2 mM L-glutamine, antibiotics/antimycotic, 100 µM 2-mercaptoethanol	Eight Achilles tendons from four mice.	RNASeq, RT-Qpcr.	Adamts16, Fgl2, Fmod, Gdf5, Itga4, Mkx, Scx, Thbs4, and Wnt10a,	The progenitor populations are indeed distinguishable by transcriptome profiling. A follow-up examination of individual colonies demonstrated that many of the pooled TP progenitor markers were quite consistently expressed amongst the individual tendon proper progenitor colonies.	Mienaltowski 2018	10.1002/jor.24076
	Rat	DMEM supplemented with 10% FBS	TDSCs and BMSCs transplantation	CCK-8 assays, qRT-PCR, Alcian-blue staining, icrosirius-red staining.	proliferative ability, Scx, AlpL, Runx2, Sox9.	TSCs are potential seed cells for tissue engineering to promote tendon-bone healing and that TSCs have a greater ability to promote tendon-bone interfacial repair than do BMSCs.	Fan 2016	10.1166/jbt.2016.1430
	Rabbits, Human	DMEM supplemented with 20% FBS.	Engineered tendon matrix (ETM) from decellularized tendon tissues.	Immunostaining, Oil Red O, Safranin O, or Alizarin Red S, qRT-PCR.	Oct-4, SSEA-1, SSEA-4, Adipogenesis, Chondrogenesis, Osteogenesis, Collagen types I and III, Tenomodulin.	ETM may be used to effectively expand TSCs in vitro and with TSCs, to enhance repair of injured tendons in vivo.	Zhang 2011	10.1016/j.biomaterials.2011.05.088
	Rat	DMEM containing 20% fetal bovine serum, 100 U/ml penicillin,100 mg/ml	chitosan/b-glycerophosphate/collagen(C/GP/Co) hydrogel	Multi-differentiation potential.	Osteogenic, Adipogenic, and Chondrogenic lineages.	Combining TSCs with C/GP/Co hydrogel significantly enhances tendon healing compared with the control, hydrogel, and TSCs groups.	Yang 2017	10.12659/MSM.906747

		streptomycin, and 2 mML-glutamine.						
	Human	LG-DMEM with 10% FBS and 1% penicillin–Streptomycin.	Biomimetic aligned poly (L-lactic acid) (PLLA) fibrous scaffold.	Fluorescence-activated cell sorting (FACS) analysis, Multipotent differentiation, SEM observation, Cell Counting KIT-8, Alkaline phosphatase (ALP), Alizarin red staining for mineralization, Real-time PCR.	CD18, CD34, CD90, CD44, CD105, Cell proliferation assay, Collagen I, Collagen III, Collagen XIV, Scleraxis, Eya 2, Elastin, Runx2, Alkaline phosphatase, Osteocalcin, Integrin α1, Integrin α5, Integrin β1, Myosin II B.	The aligned electrospun nanofiber structure provides an instructive microenvironment for hTSPC differentiation and may lead to the development of desirable engineered tendons.	Yin 2010	10.1016/j.biomaterials.2009.11.083
	Horses, Rabbits	DMEM containing 4.5 g/L glucose, 10% FBS, 100 U/ml penicillin-streptomycin, 2 mM L-glutamine, 1 mM sodium pyruvate, and 10 mM HEPES.	MSCs cultured under tenogenic (T-MSCs) and undifferentiating (U-MSCs).	Histological, Immunohistochemical, Semi-quantitative analyses.	Cell viability, Proliferation, Morphology, Matrix deposition, Type I and III collagen	Reseeding tendon matrices with U-MSCs could represent a suitable method for the functionalization of biological constructs, considering also any potential chemoattractant capability of the newly deposed extracellular matrix to recruit resident cells.	D’Arrigo 2017	10.3934/bioeng.2017.4.431

	Human	α-MEM supplemented with 2 mM glutamine, 1 % antibiotic-antimycotic mixture and 20 % fetal bovine serum.	Pulsed electromagnetic field PST	Flow cytometer, Cell differentiation, MTT assay, Wound-healing assay, Real-Time PCR.	CD9, CD73, HLA-DR, CD13, CD29, CD44, CD45, CD90, CD105, CD106, CD34, CD166, SSEA-4, Lineage Cocktail, CD18, CD146, Stro-1, CD117, Cell viability, Cell migration, Cell apoptosis, S14, Oct4, KLF4, Nanog, Tenascin C, COL1A1, VEGF.	Exposure of hTSCs to PST® did not cause any significant changes in proliferation, viability, migration, and morphology. Instead, while stem cell marker expression significantly decreased in control cells during cell culturing, PST®-treated cells did not have a significant reduction of the same markers.	Randelli 2016	10.1186/s12906-016-1261-3
	Rat	DMEM, supplemented with 10% FBS and 1% penicillin/ streptomycin.	PLGA/iron oxide microparticles	Immunocytochemistry, Cell Counting Kit-8,	SSEA4, Nucleostemin, CD90 CD44, Cytotoxicity,	The optical concentration of PLGA/IO MPs for labeling, pertaining to the cytotoxicity, and labeling efficiency of MRI and PA imaging was determined in this work. The foundation for in vivo MRI/PA dual-modal tracking of TSCs in the rat rotator cuff injury model will be laid.	Lu 2018	10.1371/journal.pone.0193362
	Rabbits	DMEM supplemented with 20% fetal bovine	Superparamagnetic iron oxide	Immuno-staining, SPIO labeling, qRT-PCR, Multi-differentiation potentials,	NS, Oct-4, SSEA-4, Viability, Proliferation,	The findings of this study show that labeling TSCs with SPIO particles is a feasible approach to track TSCs in vivo	Yang 2013	10.1007/s10439-

		serum, 100 μM 2-mercaptoethanol, 100 U/ml penicillin and 100 μg/ml streptomycin.			Adipogenesis, Chondrogenesis, Osteogenesis, PPAR, Sox9, Runx2	by MRI, which offers a noninvasive method to monitor repair of injured tendons.		013-0802-x
	rat	DMEM containing 10% fetal bovine serum, 100 U/ml penicillin, 100 mg/ml streptomycin and 2 mM L-glutamine.	CUR@MES-SiN-PP/siRNA NPs	TEM, MTT, FAM, CLSM, animal experiments,	Cell viability, macrophage polarization et al	The NPs showed dose-dependent cytotoxicity, the cell viability remained above 90% at concentrations below 10 μg/mL, and the NPs were ingested by higher cells.	Xu 2023	10.1166/sam.2023.4426
	Human	LG-DMEM with 10% FBS and 1% penicillin-streptomycin solution	3D culture microenvironments	Assessment of cell viability and proliferation, assessment of multipotent differentiation capacity, Flow cytometry analysis, Immunofluorescence staining, Cell sheet formation, Bulk RNA-seq, single-cell RNA-seq, RT-PCR, WB	cell viability and proliferation, multipotent differentiation, SCX, Egr1, FOS, BGN, DCN, LUM, ALPL, Col2A1, FABP4, SPP1, ACAN, CEBPA	3D microenvironments can regulate stem-cell function by modulating the critical cell subpopulation and identifies FGF7 as a novel regulator for tenogenic differentiation and tendon regeneration.	Zhang 2022	10.1016/j.biomaterials.2021.121238
	Human	α-MEM 10%FBS 1%antibiotic/antimitotic solution	human adipose-derived stem cells	RT-PCR, ELISA, Quantification of cell density and nuclei aspect ratio, image analysis, Immunocytochemistry	MMP-3, cell density and nuclei aspect ratio, ECM proteins, collagen	In conclusion, hASCs seem to be good candidates in modulating the behavior of native tendon cells, particularly through a balanced process of ECM	Raquel 2017	10.1002/jcp.26363

					type I, collagen type III,T enascin C	synthesis and degradation. This article is protected by copyright. All rights reserved		
	Human	DMEM-F12 with 1% kanamycin and glutamax	hMSCs secretome	Cell Viability Assessment, and Immunocytochemistry, Biomechanical Testing, Histological Analysis	viability and density, Histological and Biomechanical result	The results demonstrated that hMSCs-CM increased hTCs viability and density in vitro. Clear benefits also were observed when these primed cells were integrated into a tissue engineering strategy with an electrospun keratin scaffold.	Nuno 2017	10.1177/03635465177350
	Mice	DMEM 10%FBS a mixture of 100 U mL−1 penicillin and streptomycin	Polydimethylsiloxane	flow cytometry, Surface characterizations of PDMS substrates, Long-term cell viability	Surface characterizations, Apoptosis cell viability cell proliferation	This study demonstrated that COL/PDA coating can effectively enhance the surface biocompatibility of PDMS as verified by the enhanced adhesion and long-term proliferation of L929 fibroblasts and TSCs.	Qian 2018	10.1002/jbm.a.36254
	Human	DMEM 10%FBS and 1%penicillin/streptomycin 1% L-ascorbic acid-2-phosphate solution	RADA peptide hydrogel	TEM, CLSM, PCR, FI-AFM	TSPCs survival, apoptosis, and proliferation	the RADA-based hydrogels exert a rejuvenating effect by recapitulating in vitro specific features of the natural microenvironment of human TSPCs, which strongly indicates their potential to direct cell behaviour and overcome the challenge of cell aging and degeneration in tendon repair.	Yin 2019	10.1016/j.biomaterials.2020.119802

	Rat	DMEM 10%FBS 100 U/mL penicillin, 100 mg/mL streptomycin, and 2 mmol/L L-glutamine	Silk fibroin film	HE, RT-qPCR, Cell Viability, Immunofluorescence	COL1A1, TNC, TNMD, and SCX, surface marker expression, multilineage differentiation potential.	SF film with a bionic microstructure can serve as a tissue engineering scaffold and provide biophysical cues	Kang 2020	10.1155/2020/8865841
	Mice	low-glucose DMEM 10%FBS 1% penicillin–streptomycin	Bioinspired bimodal micro-nanofibrous scaffolds	SEM, Tensile test, Measurement of water contact angle, Immunofluorescence, Cell proliferation assay, RT-PCR, WB, ALP and Alizarin Red staining, Collagen content testing, Immunohistochemistry, TCM	EGR1,Tenomodulin ,Decorin,MKX,TGFβ1,TGFβ2,pSMAD2/ 3, Tubulin, GAPDH	micro-nanofibrous scaffolds promoted the structural and mechanical properties of the regenerated Achilles tendon. Overall, our study shows that the bimodal micro-nanofibrous scaffold devel oped here presents a promising potential to improve the outcomes of tendon tissue engineering.	Yin 2021	10.1039/d1bm01287h
	Human	DMEM 1% MEM 10% FBS, and 1% L-ascorbic acid-2-phosphate	thermosensitive BC hydrogel	qPCR, Immunofluorescence and F-actin stainings	collagen V, proteoglycan 4, lysyl oxidase, and ephrin A4	thermosensitive BC hydrogel holds great potential as an injectable cell delivery carrier of TSPCs for tendon tissue engineering.	Yin 2018	10.1088/1748-605x/aaadd1
	Rat	DMEM 10%FBS 1% penicillin–streptomycin	TSC sheet	histology, immunohistochemistry, transmission electron microscopy (TEM) and mechanical testing	TSCs characteristics TSC sheet characteristics Validation of TSC sheet implantation	cell sheets into a tendon defect significantly improved histological properties and collagen content at both 2 and 4 weeks after implan	Issei 2015	10.1016/j.actbio.2016.06.026

					TSC sheet implantation on a rat tendon defect model Assessment of TSC sheet integration	tation, indicating that TSC sheets may effectively promote tendon remodeling in the early stages of ten don healing.		
	Rat	LG-DMEM,10% FBS, and 1% penicillin–streptomycin	stem cells from human exfoliated deciduous teeth (SHED-Exos)	GelMA，GM，Exosome Adsorption and Release Assay，Histology and Immunofluorescence，Micro-CT，Scanning Electron Microscopy，Grip Test，Achilles Functional Index	SHED-Exo Proteins，Calnexin，TSG101，CD63，CD9	SHED-Exos, as natural bioactive nanoparticles, have promising translational and therapeutic potential for aging-related diseases.	Jin 2023	10.1002/adma.202211602
	Rat	LG-DMEM,10% FBS, and 1% penicillin–streptomycin	bioactive electrospun nanofiber membranes	Electrospinning，, cell proliferation, viability, adhesion and osteogenic differentiation	Col1a1，Ocn，Runx-2，Oct4	These bioactive electrospun nanofiber membranes may act as a suitable functional biomimetic scaffold in tendon-bone tissue engineering applications to enhance tendon-bone healing abilities.	Lin 2019	10.2147/ijn.s210509
	Human	None	3D-printed scaffolds	vitro engineering,Collagen fiber organization,Biomechanical evaluation	COL-I, GAG, CTGF, CTGF, TGFβ3 BMP2	The in situ tissue engineering approach shows translational potential for improving outcomes after rotator cuff repair.	Solaiman 2019	10.1088/1758-5090/ab48ca
	Human	None	hydrogel	tendon-related genes and lineage/cross	COL I, ACTA2, COMP, THBS2,	These results showed a novel strategy for directing stem cell behavior without the use of exogenous growth factors or pre-	Xu 2020	10.1039/d0

				linking genes, was obtained by implementing designer quantitative RT-PCR plates	THBS4, TNC, and TGF-β, BGN, FN, PLOD and TGM2	aligned COL I fibers, and propose that anisotropic nanocomposite hydrogels hold great potential for tendon tissue engineering applications.		bm01127d
	Human	LG-DMEM, L-glutamine penicillin streptomycin 20 and 10% FBS	Culture condition	qPCR, ELISA, Immunopotency Assays	SCX, TN-C, COL1A1, and COL3A1	these experiments allowed us to identify relevant differences in TSPCs based on culture conditions. This ability of TSPCs to acquire distinguished morphology, phenotype, gene expression profile, and functional response advances our current understanding of tendons at a cellular level and suggests responsivity to cues in their in situ microenvironment.	Carlotta Perucca 2021	10.3389/fbioe.2021.711964
	Rat	LG-DMEM 10%FBS 1% penicillin/streptomycin solution	Octacalcium Phosphate Crystals	DNA Concentration Measurement and ALP Activity Assay<br>Real-Time qPCR<br>Alizarin Red Staining<br>FTIR Analysis<br>Measurement of Ion Composition	RUNX2, Col1a1, OCN, and OPN	calcium ion (Ca2+) and inorganic phosphate (Pi) ion concentrations and pH values of the TSPCs medium. The results suggest that the difference in the osteogenic differentiation of the TSPCs is related to the ionic environment induced by OCP and CDHA, which could be related to the progress of OCP hydrolysis into CDHA. These results support the previous in vivo observation that	Liu 2023	10.3390/ijms24021235

						OCP has the healing function of rabbit rotator cuff tendon in vivo.		
	Mice	10% FBS in DMEM with 100 U/ml penicillin and 100 µg/ ml streptomycin.	mechanical loading	Cell morphology assessment and nucleostemin staining qRT-PCR microscope histochemical staining	LPL, Runx-2, and SOX-9 Runx-2, and PPARγ	MTR maintains tendon homeostasis by promoting the differentiation of TSCs into TNCs, ITR causes the onset of tendinopathy development by inducing non-tenocyte differentiation of TSCs, which may eventually lead to the formation of non-tendinous tissues in tendon tissue after long term mechanical overloading conditions on the tendon.	Zhang 2020	10.1371/journal.pone.0242640
	Rat	DMEM 10%FBS 50 µg/mL each of penicillin and streptomycin	asymmetric chitosan scaffold	CCK-8,PCR,Western blot,Histology and immunofluorescent analyses,Adhesion evaluation	GAPDH, TNMD, COL1A1	The introduction of TSPCs into the CS scaffold resulted in a synergistic effect on tendon regeneration and yielded better-aligned collagen fibers with elongated, spindle-shaped cells. These findings indicated that the application of TSPC-seeded CS scaffolds would be a feasible approach for tendon repair.	Chen 2018	10.1016/j.actbio.2018.04.027
	mice	a-MEM 20%FBS 100 mM 2-mercaptoethanol	TSPCs transplantation	Multipotent differentiation, Label-retaining cells, Western blotting FACS analysis. RT-PCR. Nucleofection and luciferase reporter assays, Histochemistry and immunohistochemistry. In vivo transplantation,	p-Smad1, Smad1 , Hsp90 and Aml-3 ,Runx2,Akp1,Spp1,Ibsp,Cebpa,Lpl,Fabp4,BGLAP1,PPARGC1A,CEBPA,CFD	These results, while offering new insights into the biology of tendon cells, may assist in future strategies to treat tendon diseases.	Bi 2007	10.1038/nm1630

				Immunocytochemistry, Microcomputed tomography analysis				
	Rat	DMEM 20%FBS 1% penicillin and streptomycin	PCL aligned nanofiber yarns	RT-qPCR, histological evaluation, Scanning electron microscopy, flow Cytometry, Live/Dead assay	CD18, CD34, CD90, CD44, CD45	electrospun bundled NFYs formed by aligned nanofibers can mimic the aligned hierarchical structure of native tendon tissue, highlighting their potential as a biomimetic multi-scale scaffold for tendon tissue regeneration.	Yang 2022	10.3389/fbioe.2022.960694
	Human	DMEM/Ham's F-12 medium with glutamine (365.3 mg/L),1 × MEM amino acids, 10% FBS and 1% L-ascorbic acid-2-phosphate	aging	FACS and immunohistochemistry<br>histological examination<br>PCR<br>ELISA<br>F-Actin Staining<br>Nuclear Staining and Cell Density Analysis<br>TUNEL<br>TEM	CD44, CD73, CD90, CD105, CD146 Musashi-1 and STRO-1 as well as negative markers CD19, CD34, CD45, HLA-DR	A-TSPCs exhibit profound deficits in forming 3D tendon tissue organoids, thus making the cell sheet model suitable to investigate the molecular mechanisms involved in tendon aging and degeneration, as well as examining novel pharmacologic strategies for rejuvenation of aged cells.	Yan 2020	10.3389/fbioe.2020.00406
	Rat	DMEM supplemented with 10% fetal bovine serum, penicil lin (100 U/mL)/streptomycin (100 mg/mL), and L-glutamine	Silk Films	scanning electron microscope<br>Biomechanical Test<br>Confocal Microscopy<br>Live/Dead Staining<br>Cell Cytotoxicity<br>RT-PCR<br>Western Blot	SCX, TNC, TNMD, and COLIA1, and activated FAK CD34,CD44,CD3,CD90	SF films with a bionic microstructure may serve as a scaffold, provide biophysical cues to alter the cellular adherence arrangement and cell morphology, and enhance the tenogenic gene and protein expression in TSPCs. FAK activation plays a key	Lu 2020	10.1155/2020/8857380

		(2 mmol/L)				role during this biological response process.		
	Rat	DMEM 10%FBS penicillin (100 U/ mL)/streptomycin (100mg/mL), and L-glutamine (2mmol/L)	SF films	MRI Histomorphometry IHC Bioinformatics Analysis Western Blot Immunofluorescence.	PI3K/AKT， TNC， TNMD， AKT， pAKT， PI3K， p-PI3K， integrin α2， GAPDH	Micropattern SF films modified by water annealing can promote remodeling of the injured tendon in vivo and regulate the tendon differentiation of TSPCs through the α2β1/FAK/PI3K/AKT signaling pathway in vitro. Therefore, they have great medical value in tendon repair.	Lu 2023	10.1155/2023/2915826
	Human	DMEM,1% MEM-Amino-acids,10% FBS and 1% L-ascorbit-acid-2-phosphate	TDSC	semi-quantitative, quantitative PCR and western blotting technologies,RT-PCR,Immunocytochemistry,Mechanical stimulation,Western blot	collagen I-binding integrins - α1, α2 and α11, the matrix metalloproteinases - MMP9, 13 and 14， lumican and versican， c-fos,HB-GAM,COMP,Decorin,Biglycan,Fibromudulin,Lumican,Versican,MMP1/2,FAK, ERK, Akt, p38 and Jnk	This study contributes to better understanding of mechanotransduction mechanisms in TPSC, which in long term, after further translational research between tendon cell biology and orthopedics, can be beneficial to the management of tendon repair.	Cvetan 2015	10.1186/s12867-015-0036-6

	Human,r abiit	LG-DMEM with 10% FBS	Type II Collagen Sponges	Immunocytochemistry Staining<br>q-PCR<br>Histology Examination	Collagen 1/2,SOX9,Aggreca n	Our study showed that CII-sponges facilitated the TSPCs to differentiate toward chondrocytes and increased the area of FCs, which suggests that CII-sponges are meaningful for the reconstruction of FC at bone tendon junction. However, the link between the two phenomena requires further research and validation.	Wang 2021	10.33 89/fce ll.202 1.682 719
	Human	DMEM 10%FBS<br>1% penicillin and streptomycin	Phosphatidylcholi ne	Dilution integrity test<br>Cytotoxicity studies<br>Scanning Electron Microscopy micrographs	Cytotoxicity biological activity	The study supported MF technology for nano-carriers fabrication and opens perspectives on the activity of PC/T3 nano-vesicles as innovative formulations for TPSCs stimulation in ECM secretion.	E.P. Lamp arelli 2022	10.10 16/j.ij pharm .2022. 12200 7
	Rat	LG-DMEM<br>10%FBS<br>100 U/ml penicillin, 100 mg/ml streptomycin and 2 mM L-glutamine	Engineered scaffold-free tendon tissue	qRT-PCR<br>fluorescence imaging<br>Ultrasound imaging<br>Biomechanical testing<br>Histology and immunohistochemistry	tendon healing.<br>Collagen<br>Tenomodulin<br>Osteocalcin	This is a proof-of-concept study demonstrating that ESFTT could be a potentially new approach for tendon repair and regeneration.	Ni 2012	10.10 16/j.bi omate rials.2 012.11 .046
	Rat	DMEM 10% fetal bovine serum and antibiotics	fibroblast-derived matrix	Immunofluorescence staining<br>Implantation of engineered tendon tissue<br>Histology<br>Mechanical examination	growth and tenogenic dif ferentiation of TSCs	These findings obtained from our study provide a basis for potential use of engineered tendon tissue containing dFM and TSCs in tendon repair and regeneration.	JIAN G 2014	10.10 16/j.jc yt.201 3.07.0 14

				RT-PCR				
	Rat	LG-DMEM<br>10% FBS and antibiotics (penicillin 100 U/ml, streptomycin 100 g/ml	MSCs and TDSC co-culture	Co-culture assay, Quantification of collagenous proteins, q-PCR, Immunofluorescence staining, In vitro cell-sheet formation, Histology and immunohistochemistry staining, Rat patellar tendon injury model, Biomechanical test	TNMD，Scx，TnC，Dcn，COL1A1，COL3A1	This study suggests that BMSCs and TDSCs co-cultured at 1:1 ratio may be an improved cell source/preparation for tendon tissue engineering.	Wu 2016	10.1089/ten.tea.2016.0248
	Rabbit	DMEM)/F12 (control)<br>or 650 μL of 1% FBS DMEM/F12+ composite scaffold	Dual-Phase Aligned Composite Scaffolds	RT–qPCR<br>TECM<br>CCK-8，live/dead staining, and morphological observation<br>Migration Assay	Col I, Col III, TNC, DCN, and TNMD	the engineered scaffold facilitates TDSC proliferation and migration, favors tenogenesis-associated gene expression, promotes tendon repair with native like hierarchically organized collagen fibers, and enhances the mechanical properties, indicating its potential value in the tendon tissue engineering field.	Ning 2022	10.1002/adtp.202200081
	Rat	RPMI 1640 supplemented with 10% FBS, 100 U/mL penicillin G, and 0.1 mg/mL streptomycin	Allogeneic TDSCs	Lymphocyte proliferation assay<br>Lymphocyte-mediated TDSC lysis assay<br>CDC assay<br>Antigen-antibody binding assay<br>Flow activated cell sorting analysis<br>Measurement of soluble immuno-modulatory factors	HRP，CD73，f MHC-II and CD86，Fas and FasL. g-IFN，TGF-b1	Allogeneic TDSCs exhibited low immunogenicity. Allogeneic TDSCs might be used for transplantation.	Pauline 2014	10.1089/ten.tea.2013.0714

	Human	α-MEM 10%FBS 1%antibiotic/antimitotic solution	human adipose-derived stem cells and TDSC co-culutre	RT-PCR, ELISA, Quantification of cell density and nuclei aspect ratio, image analysis, Immunocytochemistry	MMP-3,cell density and nuclei aspect ratio, ECM proteins,collagen type I,collagen type III,Tenascin C	In conclusion, hASCs seem to be good candidates in modulating the behavior of native tendon cells, particularly through a balanced process of ECM synthesis and degradation. This article is protected by copyright.	Raquel 2017	10.1002/jcp.26363
	Human	DMEM-F12 with 1% kanamycin and glutamax	hMSCs secretome	Cell Viability Assessment, and Immunocytochemistry, Biomechanical Testing, Histological Analysis	viability and density	The results demonstrated that hMSCs-CM increased hTCs viability and density in vitro.	Nuno 2017	10.1177/03635465177358 50
	Rat	LG-DMEM with 20% FBS and 1% penicillin–streptomycin, and 2 mM L-glutamine	Sandwich Biomimetic Scaffold	Assessments of TSPC Morphologies, F-actin Organization, and Alignment. Macroscopic and Histological Evaluation of Tendons	TSPC Morphologies, F-actin Histological et al	3D-aligned TSPCs within a biomimetic topology environment are promising for functional tendon regeneration.	Li 2023	10.1021/acsami.2c16584
	Mice	LG-DMEM with 10% FBS and 1% penicillin–streptomycin, and 2 mM L-glutamine	Histone Deacetylase Inhibitor Treated Cell Sheet	RNA Sequence, HDAC Activity Assay, FACS analysis, Differentiation Assays, IF, WB	HDAC, MKX, TNMD, Egr1, DCN, EYA2	This study uncovered an unrecognized role of HDACi in phenotypic and functional mTSPCs expansion to enhance their therapeutic potential.	Zhang 2018	10.1016/j.biomaterials.2018.03.043
	Rat	DMEM with 10%FBS, and 1% penicillin/streptomycin	Eugenol (EUG)	CCK8 Assay, Colony-Forming Assay, Apoptosis Assay, IF, RT-qPCR, WB, Animal Experiments	SCXA, TNMD, TNC, ColI (tenogenic	EUG-BMSC-EVs as a new therapeutic vehicle to facilitate TSC therapies for tendon regeneration.	Li 2022	10.1007/s00441-016-

					differentiation related genes)			2552-1
	Rat	DMEM，10% FBS, 100 U/ml penicillin，100 mg/ml streptomycin	matrix stiffness	CCK8 assay, immunofluorescences (IF), real time PCR, western blot	Tenocyte markers：THBS4, TNMD, SCX, chondrocyte marker: COL2, osteocyte markers: Runx2, Osterix, and ALP	Matrix stiffness modulated the proliferation and differentiation of TDSCs, and the regulation effect could correlate to the activation of FAK or ERK1/2.	Liu 2018	10.1038/srep22946
	Rat	LG-DMEM, 10% FBS, 50 mg/mL penicillin, 50 mg/mL streptomycin, and 100 mg/mL neomycin	dTDSC sheet	Animal Experiments, Immunochemical Staining, Biomechanical Testing, Histology, CT Imaging and Image Analysis	micro–computed tomography, histology, and biomechanical testing assess。The accumulation of iNOS1 and CD2061 cells，metalloproteinase 1 (MMP-1), MMP-13, TIMP-1	Wrapping tendon graft with a dTDSC sheet promoted graft healing after ACLR, likely via enhancing bone formation and angiogenesis by BMP-2 and VEGF, modulating macrophage polarization and MMP/TIMP expression, and physically protecting the tendon graft.	Yao 2013	10.1177/03635465221135770
	Mice	Alpha-MEM, 10% FBS, 1% of streptomycin and penicillin	Three-dimensional Uniaxial Mechanical Stimulation	Preparation of 3D tendon stem cell construct，Evaluation of the mechanical stimulated in vitro tendon-like tissue	Tenogenic markers: SCX, MKX, TNMD, COL1A1	This protocol could mimic cell differentiation in the tendon, which is helpful for the investigation of the pathological process of tendinopathy. Moreover, the tendon-like tissue is	Chen 2020	10.3791/61278

						potentially used to promote tendon healing in tendon injury as an engineered autologous graft.		
	Mice	α modified Eagle’s medium, 10% fetal bovine serum, 100 U/ml penicillin, 100 mg/ml streptomycin	3D uniaxial mechanical stimulation	Western blot, 3D bioreactor culture, RT-PCR, Histology, immunohistochemistry, Mechanical testing, Animal experiments	Tenogenic markers: Scleraxis, Mohawk, TNMD, COL1A1, Osteogenic, adipogenic, chondrogenic markers :RUNX2, ALP, CEB/P, PPARγ, SOX9, COL2A1	The importance of appropriate mechanobiological stimulation in 3D cell niches on tendon-like tissue formation and demonstrates that uniaxial mechanical loadingplays anessential role in tenogenicdifferentiationand tendonformationbyactivating thePI3K/ AKT signaling pathway	Wang 2018	10.1096/fj.201701384r
	Rat	High glucose DMEM, 20% fetal bovine serum, 100 U/mL penicillin, and 100 mg/mL streptomycin	The 532nm Laser	Biochemical assays of TDSC,Crystal Violet assay, Crystal Violet assay, Western blot, Gene chip microarray assay,Animal experiments, Hematoxylin and eosin staining, Immunohistochemistry	Genes of TDSC differentiation：Scx, Tnmd, Mkx, Dcn, PPARγ, Sox9, Runx2, tenogenic differentiation genes: Scx and Tnmd	A 532nm laser with 15 J/cm2 regulated the process of TDSC proliferation and upregulated Nr4a1 to stimulate tenogenic differentiation.	Li 2022	10.1089/photob.2022.0003
	Human	DMEM/F-12, 20% fetal bovine serum, 1% penicillin-streptomycin	Tendon-Derived Progenitor Cells	Multilineage Differentiation, Cell Isolation and Expansion	Adipogenesis, Osteogenesis, Chondrogenesis markers: ALP, SOX9, Adiponectin	The promise of patellar tendon tissue as a source of progenitor cells for use in biologic therapies for the treatment of tendinopathy.	Leonardi 2021	10.1177/23259671211023452

	Rat	Dulbecco's modified Eagle's medium, 10% fetal bovine serum, 1% penicillin-streptomycin antibiotic	TGFβ1 over expression	CCK-8, Immunofluorescence analysis, Differentiation capacity analysis, overexpressing TGFβ1, Western blotting, Histological examination	Fibrogenic and chondrogenic markers: collagen type II, α-SMA, p-smad2, aggrecan,	TGFβ1-TSC therapy may be a candidate for effective tendon fibrosis.	Yu 2022	10.1186/s13018-022-03241-y
	Human	a-MEM supplemented with 10% FBS and 1% penicillin-streptomycin-neomycin	Inhibition of IKKb/NF-kB and AuNC-siRNA	Flow cytometry analysis, Multipotent differentiation, b-gal staining, IF, RT-PCR, WB, animal experiments, Biomechanical testing	Senescence marker, Tenogenic potential, IKKb/NF-kB pathway,	This study provides a promising therapeutic strategy for degenerative RCT via intra-articular delivery of IKKb siRNA using AuNCs.	Wang 2022	10.1016/j.omtn.2021.12.026
	Human	LG-DMEM supplemented with 10% FBS	TGF-β3 and BMP-2 and collage II sponge	Multidifferentiation, RT-PCR, IF, IHC	Col1α1 Col2α1 Tenascin C Sox 9 GAPDH	TSPCs are able to differentiate into fibrocartilage-like cells and thus might well be one potential cell source for fibrocartilage regeneration in a damaged BTJ repair.	Qin 2020	10.1016/j.jot.2019.08.006
	Rabbit	HG-DMEM, supplemented with 10% FBS and 50 µg/ml gentamycin	29-mer peptide	Animal experiment, Colony-forming, Immunocytochemistry, Biomechanical testing, BrdU, WB, RT-PCR, IHC, Histological	Oct4 , nestin, Col1a1, Col3a1 Tenascin C Mkx, Egr1, CD146, Histological	This study analysis of TSPC populations in the wound healing process supports the hypothesis that substantial expansion of resident TSPC by exogenous growth factor is beneficial for tendon healing	Ho 2019	10.1186/s13287-018-1110-z

	Human	DMEM with 10% FBS, and 1% penicillin-streptomycin	Decellularized Porcine Tendon	Histological Staining and Immunohistochemistry, Determination of Collagen Content, TEM, Mechanical Testing	Mechanical result, Histological score, COLI, Collagen Content	These findings suggest a promising strategy for functional tendon tissue regeneration and further studies are warranted to develop a functional tendon tissue regeneration utilizing tendon stem/progenitor cells integrated with a tendon-derived decellularized matrix.	Song 2018	10.117<br>7/096<br>36897<br>18805<br>383
	Rat	DMEM containing 10% FBS and 1% P-S	AgNPs	MTT viability assay, Cellular morphological change, Annexin-V-GFP and PI apoptotic assay, JC-1 mitochondria membrane potential (MMP) assay, CM-H2DCFDA cellular oxidative stress detection assay, apoptotic assay, Colony forming assay, qRT-PCR,	Cell viability assay, apoptotic activity, Tnmd, beta-actin, AgNPs concentration	AgNPs are not a good scaffolding coating material for tendon engineering.	Cheun<br>g<br>2015	10.10<br>39/c5t<br>x0034<br>9k
	Rat	0.5 µM dexamethasone, 50 µM indomethacin, 50 µM isobutylmethylxanthine (IBMX) and 10 µg/ml insulin	decellularized tendon hydrogel	Colony formation，flow cytometry analysis；Immunofluorescent；ELISA；CCK-8 assay；H&E staining；Masson’s Trichrome staining；RT-qPCR；	Colony formation, GAPDH, SCX, TNMD, TNC, COLI, COLII, CD73, CD90, CD105, CD34, CD45, Nanog, Oct-4, SSEA-1,	These findings indicated that the T-gel, with its retained nanofibrous structure and some bioactive factors of native tendon ECM microenvironment, represents a promising hydrogel for tendon regeneration.	Liang-<br>Ju<br>Ning	10.33<br>89/fce<br>ll.202<br>1.651<br>583

	Rat	LG-DMEM with 10% FBS	TDSC and SIS Hydrogel	cell adhesion, proliferation, and tenogenic differentiation, animal experiment	cell adhesion and tenogenic differentiation, tendon regeneration and antiadhesion capacity, CD163,CD68	This study showed that biologically prepared SIS scaffolds synergistically promote tendon regeneration with TDSCs and achieve antiadhesion through M2 polarization of macrophages.	Mao 2022	10.3390/cells11172770
	Human	α-MEM with 10% FBS and 1% AB/AM	continuous and aligned electrospun nanofiber threads	Morphological Characterizations, animal experiment, Alamar Blue assay, H&E staining, IF,	Mechanical Properties, ColI, Col III, TNC, SCX,	This study obtained clearly indicate that key features of the developed scaffold, mimicking native tissue, are crucial for the development of tendon tissue engineeringed substitutes.	Laranjeira 2017	10.1002/smll.201700689
	Rat	DMEM-LG supplemented with 10% FBS, 100 U/mL penicillin, and 100 μg/mL streptomycin	BMSC and TDSC transplantation	Animal experiment, Biomechanical Test, Multilineage differentiation potential, HE, IHC, IF	CD29, CD44, CD90, TenC, CM-Dil, Biomechanical result,	Compared with BMSCs, TDSCs showed higher regenerative potential while treating ruptured Achilles tendons in rats.	Al-Ani 2015	10.1155/2015/984146
	Rat	DMEM/F12 (1:1) medium containing 20% FBS and 1% penicillin and streptomycin	Different loading regimens of cyclic tensile strain	Cell Proliferation,RT-PCR, Transcriptome Microarray Analysis	Cell Proliferation, GAPDH, SCX, TNMD, Ten-C, Col1	The custom-designed 3D tensile bioreactor used in our study provides testimonies that cyclic tensile strain with different parameters has different effects on the proliferation and tenogenic differentiation of TDSCs; cyclic tensile strain with 0.5 Hz at 4% amplitude may	Xu 2015	10.1155/2015/790804

						be the optimal condition for the proliferation and tenogenic differentiation of TDSCs		
	Mice	LG-DMEM with 10% FBS	parallel microgrooved polydimethylsiloxane (PDMS) membrane	multiple lineage differentiation, flow cytometry, RT-PCR,	Tri-lineage differentiation assays, CD34, CD44, CD90.2, CD73, CD105, CD146, SSEA4, Sca-1, Nanog, Sox2, Oct4, Tnmd, SCX, Col1, Col3, Col6, DCN, TNC	simulation of native tendon structure via using parallel microgrooved topography can promote mTDSC differentiation specifically towards tenogenic lineage and prevent non-tenogenic lineage differentiation, providing an insight into the design of tendon regenerative materials.	Shi 2017	10.1088/1748-605X/12/1/015013
	Rat	DMEM containing 10% FBS and 1% PenicillinStreptomycin	KGN@MBGs and hydrogel	Animal experiment, Micro-CT analysis, Fibrocartilage layer staining, Biomechanical test, WB	CD31, CD45, CD90, CD105, Col1, Runx2, BMP2, GAPDH, COlII	The bioactive agents-loaded hydrogel reported in this study is a valuable addition to the arsenal of biomaterials in applications to chronic tendon-bone junction injuries.	Huang 2022	10.1016/j.mtchem.2021.100720

Supplementary Table 2: Top 10 Country with Most Publications

Country	Article	Citations	Citations/Article
China	211	5356	25.5
USA	69	4145	60.1
Germany	18	611	33.9
Italy	15	266	17.7
England	9	306	34.0
Japan	8	382	47.75
South Korea	7	78	11.1
Switzerland	6	179	29.8
Australia	6	75	12.5
Portugal	6	224	37.3

Supplementary Table 3: Top 10 Most Productive Journal of TDSCs

Rank	Journal	Publications	Citations	Impact Facotr (2022)
1	Stem Cells International	19	243	4.3
2	Journal of Orthopaedic Research	14	1000	2.8
3	Stem Cell Research & Therapy	14	404	7.5
4	Biochemical and Biophysical Research Communications	11	224	3.1
5	Frontiers in Cell and Developmental Biology	11	113	5.5
6	Cellular Physiology and Biochemistry	9	267	None
7	Tissue Engineering Part A	9	561	4.1
8	Plos One	8	286	3.7
9	American Journal of Sports Medicine	7	399	4.8
10	Biomaterials	7	763	14

Supplementary Table 4: Top 20 Keyword related TDSCs

Keyword	Occurrences	Rank	Keyword	Occurrences	Rank
Tendon Stem Cells	142	1	In-vitro	54	11
Regeneration	110	2	Bone-Marrow	41	12
Stem Cell	108	3	Gene	37	13
Differentiation	107	4	Achilles Tendon	36	14
Expression	86	5	Injury	35	15
Mesenchymal Stem Cells	80	6	Osteogenic Differentiation	34	16
Tendinopathy	64	7	Tendon Regeneration	33	17
Extracellular Matrix	59	8	Tendon	31	18
Proliferation	59	9	Tenogenic Differentiation	30	19
Matrix	55	10	Bone	28	20
